# Smart Cargo Delivery System based on Mesoporous Nanoparticles for Bone Disease Diagnosis and Treatment

**DOI:** 10.1002/advs.202004586

**Published:** 2021-03-16

**Authors:** Panpan Pan, Qin Yue, Juan Li, Meiqi Gao, Xuanyu Yang, Yuan Ren, Xiaowei Cheng, Penglei Cui, Yonghui Deng

**Affiliations:** ^1^ Department of Chemistry Department of Gastroenterology Zhongshan Hospital of Fudan University State Key Laboratory of Molecular Engineering of Polymers Fudan University Shanghai 200433 China; ^2^ Institute of Fundamental and Frontier Sciences University of Electronic Science and Technology of China Chengdu 610051 China; ^3^ Department of Orthopedic Surgery Xin Hua Hospital Affiliated to Shanghai Jiao Tong University School of Medicine Shanghai 200092 China; ^4^ State Key Lab of Transducer Technology Shanghai Institute of Microsystem and Information Technology Chinese Academy of Sciences Shanghai 200050 China

**Keywords:** bone targeting, diagnosis and treatment, mesoporous nanoparticle, smart cargo delivery, stimuli responsiveness

## Abstract

Bone diseases constitute a major issue for modern societies as a consequence of progressive aging. Advantages such as open mesoporous channel, high specific surface area, ease of surface modification, and multifunctional integration are the driving forces for the application of mesoporous nanoparticles (MNs) in bone disease diagnosis and treatment. To achieve better therapeutic effects, it is necessary to understand the properties of MNs and cargo delivery mechanisms, which are the foundation and key in the design of MNs. The main types and characteristics of MNs for bone regeneration, such as mesoporous silica (*m*SiO_2_), mesoporous hydroxyapatite (*m*HAP), mesoporous calcium phosphates (*m*CaPs) are introduced. Additionally, the relationship between the cargo release mechanisms and bone regeneration of MNs‐based nanocarriers is elucidated in detail. Particularly, MNs‐based smart cargo transport strategies such as sustained cargo release, stimuli‐responsive (e.g., pH, photo, ultrasound, and multi‐stimuli) controllable delivery, and specific bone‐targeted therapy for bone disease diagnosis and treatment are analyzed and discussed in depth. Lastly, the conclusions and outlook about the design and development of MNs‐based cargo delivery systems in diagnosis and treatment for bone tissue engineering are provided to inspire new ideas and attract researchers’ attention from multidisciplinary areas spanning chemistry, materials science, and biomedicine.

## Graphical abstract

Tailored mesoporous nanoparticles‐based cargo delivery systems with abundant compositions and unique structures exhibit unprecedented drug‐loading capacity, stimuli‐responsive (e.g., pH, photo, ultrasound) controllable delivery, and specific bone‐targeted therapy, and excellent structure enables cargo sustained release, providing a novel avenue for bone disease diagnosis and treatment.

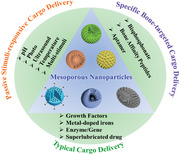

## 1 Introduction

Bone diseases, such as bone cancer (e.g., osteosarcoma, bone metastasis), bone infection, osteoporosis, osteoarthritis (OA), and nonunion defects constitute a major issue that threatens public health as a consequence of progressive aging of bone.^[^ ^1^ ^]^ Autografts and allografts are regarded as the most traditional treatment strategies for bone reconstruction.^[^ ^2^ ^]^ Most pathologies can be treated in clinical treatment, but some of these treatments may present several drawbacks.^[^ ^3^ ^]^ Biocompatible targeted nanoparticles have emerged as an effective vehicle to transfer cargo for targeted delivery, on‐demand release, and reduce side effects. In particular, mesoporous nanoparticles (MNs, 2–50 nm in one or more dimensions) have attracted increasing attention in maximizing the content of cargo encapsulated, minimizing premature release, and targeted releasing the cargo due to their well‐defined and tunable size/shape‐dependent physicochemical properties for immobilizing targeting ligands and guest species, as well as high surface‐to‐volume ratio for loading various of biomolecules and therapeutic agents.^[^ ^4^ ^]^

Given that bone matrix including inorganic minerals (e.g., hydroxyapatite, colloidal calcium phosphate) and organic matrices (e.g., Type I collagen) that are nanosized self‐assembly, MNs could absorb into the tissue microenvironment for repair diseased osseous tissue.^[^ ^5^ ^]^ Among all kinds of inorganic MNs, mesoporous silica nanoparticles (*m*SiO_2_, MSNs) have attracted tremendous interest owing to their exceptional merits such as high specific surface area and large pore volume, different mesoporous channels, tunable sizes and shapes, excellent biocompatibility as well as ease of surface functionalization.^[^ ^6^ ^]^ Particularly, silicon involved in the process of bone calcification could stimulate new bone growth by initiating bone matrix mineralization or promoting bone cell activity, displaying great potential in osteogenesis and angiogenesis. Besides, extensive mesoporous nanoparticles, such as mesoporous calcium phosphate (*m*CaP),^[^ ^7^ ^]^ mesoporous bioactive glass (*m*BG),^[^ ^8^ ^]^ mesoporous calcium‐silicate (CaSiO_3_), mesoporous carbon,^[^ ^9^ ^]^ mesoporous hydroxyapatite (*m*HAP),^[^ ^10^ ^]^ mesoporous polydopamine (*m*PDA),^[^ ^11^ ^]^ *m*TiO_2_,^[^ ^12^ ^]^ *m*ZrO_2_,^[^ ^13^ ^]^ and silicate,^[^ ^14^ ^]^ also receive considerable attention in skeletal‐related disorders diagnosis and treatment though different strategy such as targeted and controlled drug/protein/gene vehicles, bioimaging, as well as bone tissue engineering. Moreover, metal‐doped (e.g., Cu, Eu, Zn, Sr, Co, Ga) or core–shell (yolk‐shell) or heterogenous MNs endow nanomaterial multifunctional possibility to obtain improved therapeutic effects. It is urgent to explore the bone disease diagnosis and treatment application of innovative materials by taking full advantageous features of MNs.

**Scheme 1:**
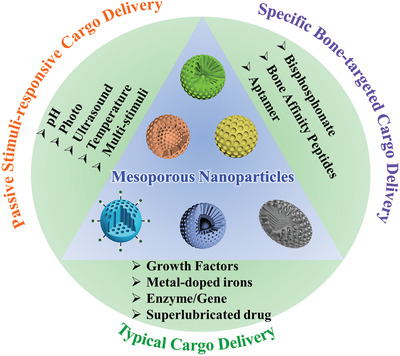
Various smart cargo delivery systems based on mesoporous nanoparticle for bone disease diagnosis and treatment.

In this review, the recent development proceeding of diverse MNs in bone disease diagnosis and treatment in past decades is discussed in depth (**Scheme** **1**). First, the properties of diverse MNs and their therapy and diagnosis mechanisms for bone‐related diseases are elucidated in detail, which is the foundation and plays a crucial role in the design of MNs. Additionally, the relationship between MNs‐based multifunctional nanocomposites and bone regeneration, as well as the mechanisms among the distinct structure of MNs and cargo sustained release are summarized. Especially, this review discusses and emphasizes the different therapy and diagnose strategies such as typical cargo sustained release, controllable and stimuli‐responsive (e.g., pH, photo, ultrasound, and multistimuli) cargo delivery, specific bone‐targeted therapy, and effective imaging agents for their application in skeletal‐related disorders diagnosis and treatment. Lastly, the conclusions and outlook related to the design and development of MNs in diagnosing and treatment for bone tissue engineering are outlined.

## 2 Types of Mesoporous Nanoparticles

### 2.1 Mesoporous Silica Nanoparticles (*m*SiO_2_)

Among all the available MNs, MSNs are the new candidate as smart drug delivery vehicles to the field, owing to their unique and superlative properties.^[^ ^15^ ^]^ Silicon is a vital element in the mechanism of biomineralization and plays a crucial role both in bone growth and calcification.^[^ ^16^ ^]^ Schwarz^[^ ^17^ ^]^ identified a close relationship between the silicon element and collagen. During the formation of cross‐links between proteoglycans and collagen, silicon not only stabilizes bone matrix but also inhibits their degradation in vivo. MSNs have been acted as a vivid candidate for an efficient cargo vehicle in bone reconstruction research owing to tremendous advantages, such as excellent biocompatibility, the intrinsic stable siliceous frameworks, and excellent mechanical strength, large surface area, and regular mesoporous channel endowing them storage capability as well as high thermal stability.^[^ ^18^ ^]^ More importantly, compared with other MNs, MSNs not only possess easily functional surfaces due to a large amount of surface hydrophilic silanol groups but also function as a protective layer against enzymatic/pH‐mediated cargo degradation in vivo. In this regard, Wu and co‐workers review systematically the synthetic method of MSNs with nanoarchitecture structure and surface functionality.^[^ ^6b^ ^]^

**Figure 1:**
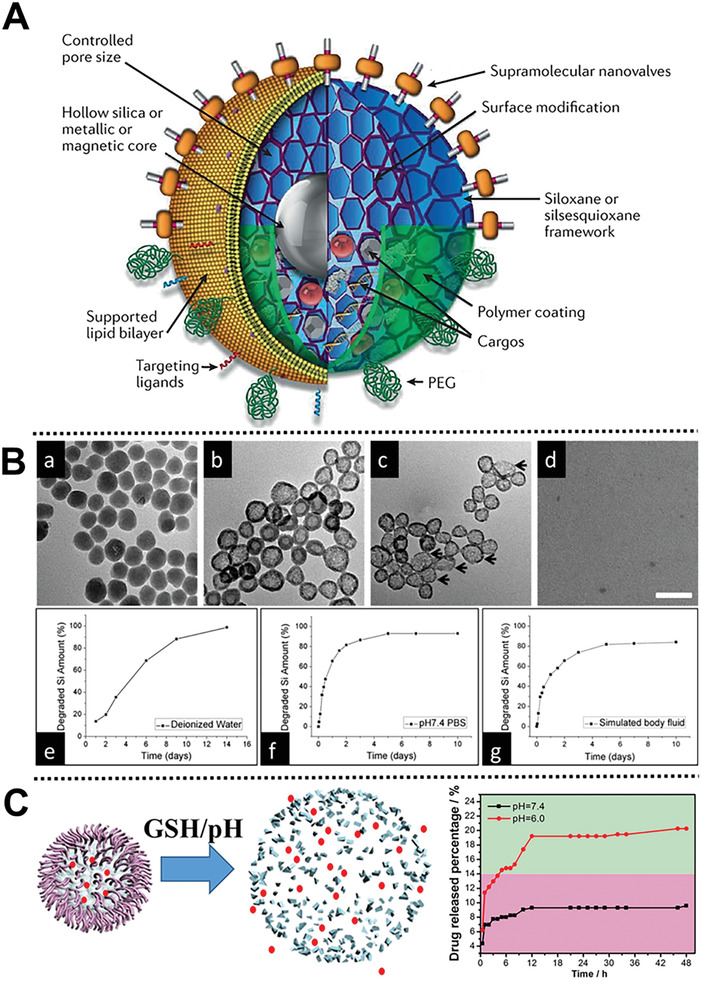
A) Schematic illustration of the modular features of MSNs and silsesquioxane‐based delivery platforms. Reproduced with permission.^[^ ^26^ ^]^ Copyright 2020, Nature. B) Carrier decomposition and degraded amount of MSNs‐drug composites. Reproduced with permission.^[^ ^28^ ^]^ Copyright 2013, American Chemical Society. C) Scheme of the biodegradation process and drug release profiles of DOX‐PEG/Mn‐HMSNs. Reproduced with permission.^[^ ^19a^ ^]^ Copyright 2016, American Chemical Society.

In general, ordered MSNs are synthesized through the surfactant‐templating approach, which predominantly utilizes small cationic surfactants (e.g., cetyltrimethylamonium bromide, CTAB) and amphiphilic block copolymer (e.g., amphiphilic triblock PEO_106_‐PPO_70_‐PEO_106_ copolymers, Pluronic F127; amphiphilic triblock PEO_20_‐PO_70_‐PEO_20_ copolymers, Pluronic P123, etc.) as structure‐directing templates and tetraethoxysilane (TEOS) or organosilica (e.g., 1,2‐bis(triethoxysilyl)‐ethane, BTEE) as the silica source. For example, the Shi group has obtained MSNs through the small molecular template (CTAB) and organosilane (chlorotrimethyl silane)‐directed assembly method and investigated their latent applications in cargo delivery and tumor therapy.^[^ ^19^ ^]^ Besides, integration of the magnetic core can endow the particle unique magnetocaloric effect and expand the applications in magnetic hyperthermia and magnetic resonance imaging.^[^ ^20^ ^]^ Our group reported a series of facile and effective strategies to construct a highly ordered core–shell magnetic MSNs with large pore size and open mesoporous channels through biliquid‐phase interface coassembly using small cationic surfactants CTAB, or amphiphilic block copolymers‐directed interface coassembly.^[^ ^21^ ^]^ Moreover, compared with the calcination method, the solvent extraction method could retain abundant functional groups on the surface of MNs to more potential and wide application. Moreover, more efforts were paid to develop surface modifications of MNs to design stimuli‐responsive nanovehicles or to enhance the bone targeting ability.^[^ ^6^, ^22^ ^]^ Most recently, despite tremendous studies around MSNs on the biomedical application, MSNs are often considered to be toxic regardless of their source. Even though the silica is “generally regarded as safe” by the US Food and Drug Administration (FDA), it still meets great bottlenecks in practical clinical applications.^[^ ^6^, ^23^ ^]^ Many previous studies proved that small MSNs possess relatively good biocompatibility.^[^ ^24^ ^]^ In a recent review, Brinker and co‐workers pointed out that not all amorphous silica nanoparticles are toxic regardless of structure (e.g., similar size, shape,^[^ ^25^ ^]^ element doping, composition, surface chemistry, and surface charge), and tremendous work has been done to explore how composition as well as synthesis affect biotoxicity (**Figure** **1**A).^[^ ^26^ ^]^ For instance, Zhang et al. systematically explored the toxicity of the amorphous MSNs depend on processing pathway (colloidal vs pyrolytic).^[^ ^27^ ^]^ Huang et al.^[^ ^25a^ ^]^ designed fluorescent MSNs with two aspect ratios (1.5, 5) for detecting the biosafety and clearance in vivo. It was found that particle shape of MSN significantly affected on its organ distributions, and short‐rod MSNs display a faster clearance rate than long‐rod MSNs. The relationships between structure and toxicity and the relationships between synthetic methods and material structure were discussed in detail by Brinker et al.^[^ ^26^ ^]^

Besides the biocompatible component, biodegradable moieties can also be introduced to achieve controlled release of cargo, for instance, introducing organic groups into the siloxane framework.^[^ ^15c^ ^]^ The adjustable degradation rate and mechanism could also be of vital importance for the design of safe and biodegradable MSN‐drug composite nanoparticles for simultaneous drug delivery and silica decomposition. Inspired by this concept, Li and co‐workers designed a special type of MSNs‐cargo nanocomposites for controllable cargo release and simultaneous nanocarrier decomposition.^[^ ^28^ ^]^ The release of cargo molecules triggers the nanocarrier decomposition, which starts from the center of MSNs, results in its complete fragmentation, and eventually eases to eliminate via renal systems (Figure 1B). Shi group creatively designed polyethylene glycol (PEG)‐modified manganese (Mn)‐doped hollow MSNs (Mn‐HMSNs) to fast promote the biodegradation and theranostic functions of Mn‐HMSNs under mild acidic conditions, further accelerating the breaking of Si—O—Si bonds (Figure 1C).^[^ ^19a^ ^]^ The fast biodegradation of this nanovehicle accelerated cargo release and enhanced T1‐weighted magnetic resonance imaging of bone tumor. This strategy provides a novel and simple “metal ion‐doping” avenue to open up a door for on‐demand biodegradation of cargo nanocarriers.

### 2.2 Mesoporous Hydroxyapatite Nanoparticles (*m*HAP)

Hydroxyapatite (Ca_10_(PO_4_)_6_(OH)_2_, HAP), a dominant position in the inorganic matrix of natural bone, has aroused essential attention over other carriers in large bone regeneration owing to its unique merits such as biocompatible, nontoxic, bioactivity, noninflammatory, and mechanical capacities.^[^ ^29^ ^]^ As a bioactive ceramic, HAP with a similar mineral component of bone shows excellent biocompatibility, bioactivity, osteoconductivity, and osteoinductivity.^[^ ^30^ ^]^ *m*HAP plays an irreplaceable role in bone tissue engineering owing to the hierarchical porous architecture and high surface area. Moreover, it can be a potential drug delivery nanosystem for loading proteins and prolonging their release by desorption and migration via the mesopores.^[^ ^31^ ^]^ Up to now, great efforts have been paid to improve the cargo loading capacity by modifying many organic groups (e.g., carboxylic acid, sulfonic acid) on the surface of *m*HAP.^[^ ^32^ ^]^

### 2.3 Mesoporous Calcium Phosphate Nanoparticles (*m*CaPs)

CaPs, as potential inorganic nonmetal biomaterial, mainly exist in nature and vertebrate bones. It plays an essential role in bone regeneration because it could enhance osteoinduction and osteopontin synthesis.^[^ ^33^ ^]^ *m*CaPs are regarded as a promising nanovehicle for cargo delivery and bone regeneration owing to their unique bioactivity as well as a similar inorganic matrix of bone tissue. Some biomolecules (e.g., nucleic acids) with high biocompatibility were introduced to the synthesis of *m*CaPs to tune their surface and structure parameter.^[^ ^7^ ^]^ In the synthesis of *m*CaPs, some biomolecules can not only supply the phosphorus source but also hinder disordered growth via chelating with calcium ions.

### 2.4 Mesoporous Bioactive Glass Nanoparticles (*m*BGs)

Among these bioactive inorganic materials, bioactive glass nanoparticles have exhibited extensive potential in bone tissue engineering owing to their merits of distinct structure and composition, as well as bonding directly with bone tissue via an apatitephase.^[^ ^34^ ^]^ *m*BGs as biocement were regarded as a sign of osteoinductivity along with excellent osteoconduction and bone matrix formation as a comparison with the conventional calcium phosphate cement.^[^ ^35^ ^]^ Biodegradable *m*BGs have possessed wide applicability for cargo delivery and bone tissue regeneration.^[^ ^34^, ^36^ ^]^ Tailoring bioactive inorganic materials into the mesoporous structure can significantly improve theirs in vitro bioactivity.^[^ ^37^ ^]^ For example, the existence of mesopores in the BG matrix greatly accelerates the deposition rate of apatite, as demonstrated by Zhao et al.^[^ ^38^ ^]^ More efforts have been paid to design diverse functionalized *m*BG‐based nanocomposites to stimulate angiogenesis, which include the manipulation of angiogenic growth factors, as well as mimicking hypoxic conditions.^[^ ^8^, ^39^ ^]^ Most interestingly, the synergism of *m*BGs and controllable amount of mineral elements (e.g., Cu, Sr, Mg) has been recognized as an effective approach to impart antibacterial activity and stimulation of osteogenesis.^[^ ^40^ ^]^

## 3 MNs as Typical Cargo Sustained Release Systems

MNs have exhibited extensive potential in cargo sustained release systems owing to their unique advantages of large surface area, large pore volume for high cargo capability, easy functional surface, and tunable pore size.^[^ ^41^ ^]^ In 2001, mesoporous silica nanoparticles (e.g., MCM‐41) were successfully synthesized via using cationic surfactant (C_16_TAB and C_12_TAB) as a template and first acted as a drug storage and release system.^[^ ^42^ ^]^ In recent years, many methods have been designed to fabricate functional MNs to further increase the loading capacity of cargo and the sustained release period.^[^ ^43^ ^]^ The applications of MNs for cargo delivery and controlled release in bone disease treatment are summarized in **Table** **1**.

**Table 1:** Examples of MNs as nanocarriers for cargo delivery and controlled cargo release for bone disease treatment

MNs‐based cargo delivery systems	Template	Precursor	Pore size [nm]	Surface area [m^2^ g^−1^]	Cargo loaded	Loading capacity [µg mg^−1^]	Release rate/days	Cell line in vitro	Refs.
MSNs‐pep	CTAB	TEOS	/	/	BMP‐2/DEX	1.7	61.3%/27	BMSCs	^[^ ^45a^ ^]^
MSNsPPR/pDNA	CTAB	TEOS	2.7	621.6	BMP‐2 pDNA/DEX	20:1	75.0%/20	BMSCs	^[^ ^49^ ^]^
MSN Rods	P123	TEOS	7.3, 20	496	BMP‐2	279	70%/3	RAW264.7, BMSCs	^[^ ^51^ ^]^
TGF‐*β*@Cu‐*m*HAPs	/	CaCl_2_, Creatine phosphate	14.1	88.5	DOX, miR‐10a IL‐2/TGF‐*β*	113.8	74.64%/0.5	BMSCs	^[^ ^52^ ^]^
Sr‐*m*CaPs	/	FBP, CaCl_2_, SrCl_2_·6H_2_O	17.2	110.1	Vancomycin	30.3 ± 0.6	83.5 ± 2.0%/ 2.5	rBMSCs	^[^ ^10^ ^]^
MSNs	CTAB	TEOS	2.2	163.3	DMOG		≈91%/14	hBMSCs	^[^ ^58^ ^]^
5% Cu‐MSNs	CTAB	TEOS	2.3	218.4	/	/	77% Si, 80% Cu/21	RAW 264.7	^[^ ^54^ ^]^
MSN‐CC‐PEI	CTAC	TEOS	15	189.8	HAS2	420	69%/0.5	OA synoviocytes	^[^ ^71^ ^]^
MSNs‐NH_2_@PSPMK	CTAB	TEOS	/	299.1	DS	17.4	59.8%/3	Chondrocytes	^[^ ^74^ ^]^
MSNs‐NH_2_@PMPC	CTAB	TEOS	5.0	50	DS	39	40.6%/3	Chondrocytes	^[^ ^77^ ^]^
MSN@CaP‐HA	CTAB	TEOS	2.9	1320	DOX	30.7	82%/1	MDA‐MB‐231, NIH3T3 cells	^[^ ^82^ ^]^
Chitosan@MSNs	CTAB	TEOS	2.3	547.9	DEX/BMP‐2	35/0.248	92%/87%/1	BMSCs	^[^ ^84^ ^]^
MSNs‐ISL	CTAB	TEOS	4.2	807.2	ISL	157.6	42.02% ± 1.63%/2.5	BMMs	^[^ ^89^ ^]^
CPPA	/	CaCl_2_, ADP/AMP	≈4.1	55.2	DOX	127	42.3%/5	hBMSCs	^[^ ^91^ ^]^
SIP@C1Sph	CTAB, F127	TEOS, Sucrose	3.2	1650	Ru dye	571	20%/2	HOS	^[^ ^96^ ^]^
ConA‐grafted MSN_ATU_	CTAB	TEOS	2.4 ± 0.1	1210 ± 35	DOX	117	71%/1	MC3T3‐E1, HOS	^[^ ^97^ ^]^
Ce6@ZA/MSN	CTAB	TEOS	/	/	DOX‐TK‐DOXY	123, 51	76.89%, 78.34%/6	OS‐732	^[^ ^103^ ^]^
UCNPs@SiO_2_@*m*SiO_2_/Ca	C_18_TMS	TEOS	3.8	485	ZnPc	500	26.5%/7	BMSCs	^[^ ^108^ ^]^
RGD–Bi_2_S_3_@MSN	CTAC	TEOS	6	273.89	DOX+NIR	499.3	30%/10 min	UMR‐106	^[^ ^115^ ^]^
Fe(VI)@HMON‐PpIX	CTAC	TEOS	/	227.9	LA	191	/	Saos‐2	^[^ ^121^ ^]^
MHA‐PNIPAAM	CTAB	CaCl_2_, K_2_HPO_4_	4	/	SIM	7.7	≈70%/16	BMSCs	^[^ ^31a^ ^]^
pep@MSNs‐RA	P123	TEOS	4.95	292.41	BFP‐1 peptide	4.84 × 10^–3^	≈90%/35	hMSCs	^[^ ^123^ ^]^
Fe_3_O_4_@SiO_2_@*m*SiO_2_@ crown ethers	CTAB	TEOS	3.2	405 ± 1	DOX	470	22%/80 min	L929 cell	^[^ ^124^ ^]^
BGN@*m*PDA	/	SiO_2_, CaO, P_2_O_5_, Dodecylamine	/	/	DOX	590	≈10%/45	MC3T3‐E1	^[^ ^126^ ^]^
MSN‐ALN‐Gd	CTATos	TEOS	/	11.5145	DEX	500	≈62%/ 22	BMSCs, HUVEC, MC3T3‐E1	^[^ ^131^ ^]^
Au@MSNs	CTAB	TEOS	2.4	176.06	ZOL	115.8	≈64%/1	MDA‐MB231 cells	^[^ ^134^ ^]^
Gd‐UCNP@MS‐ZA‐PAA	CTAC	TEOS	/	/	PL	226.3	86.17 ± 2.17%/4	BMMs, 4T1, / RAW264	^[^ ^137^ ^]^
MSNs@PEI	CTAB	TEOS	1.35	427	SiGLO, Osteostatin	31.25	≈98%/2	MEFs	^[^ ^144^ ^]^

### 3.1 Growth Factors for Osteogenic or Angiogenic Differentiation

**Figure 2:**
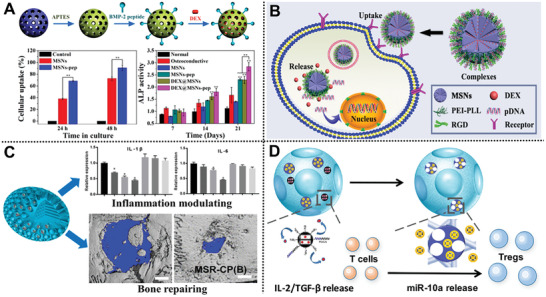
A) Scheme for the preparation and osteoinductive ability of DEX@MSNs‐pep nanoparticles. Reproduced with permission.^[^ ^45a^ ^]^ Copyright 2015, American Chemical Society. B) MSNs‐BMP‐2/DEX codelivery system for osteogenic differentiation.^[^ ^49^ ^]^ Copyright 2019, American Chemical Society. C) BMP‐2/MSNs rods for bone regeneration. Reproduced with permission.^[^ ^51^ ^]^ Copyright 2020, Springer. D) Scheme of functionalized PLLA NF‐SMS codelivery system. Reproduced with permission.^[^ ^52^ ^]^ Copyright 2018, American Chemical Society.

The bone formation and remodeling process can be tuned by growth factors, local production of cytokines, as well as the systemic hormones.^[^ ^44^ ^]^ Most of the recent studies have direct incorporation or the surface functionalization with growth factors such as bone morphogenetic proteins (BMPs, such as BMP‐2, BMP‐6, BMP‐7, and BMP‐9),^[^ ^45^ ^]^ vascular endothelial growth factor,^[^ ^46^ ^]^ transforming growth factor *β* (TGF‐*β*), insulin‐like growth factor, as well as platelet‐derived growth factor into MNs to stimulate the osteogenic and angiogenic differentiation of stem cells, further promoting bone regeneration. Especially, recombinant human BMP‐2 has been proved as extremely effective and the most potent osteoinductive growth factors in inducing new bone formation in vivo.^[^ ^47^ ^]^ However, the treatment of BMP‐2 may cause some adverse effects, such as immunological reaction and short half‐life. By introducing the BMP‐2 gene/peptide into MN, it enables continuous expression of BMP‐2.^[^ ^48^ ^]^ Based on this, the He and his co‐workers report a novel functionalized dexamethasone (DEX)‐loaded MCM‐41‐type MSNs via a surfactant‐based method by covalent conjugation of BMP‐2 peptide and aminated MSNs to construct osteogenic nanocarriers (DEX@MSNs‐pep) (**Figure** **2**A).^[^ ^45a^ ^]^ This nanocarrier synergistically promoted the osteogenic differentiation of bone marrow mesenchymal stem cells (BMSCs) in vitro, showing potential applications in bone tissue engineering.

Inspired by synergistic bioactivity of dual factors, synthesized polycation (polylysine‐modified polyethyleneimine copolymer, PEI‐PLL‐25k) and arginine‐glycine‐aspartate (RGD) peptide‐functionalized MSNs were successfully fabricated by He and co‐workers to construct a novel osteogenic nanocarrier, which effectively realizes the dual‐delivery of plasmid DNA (BMP‐2 gene) and DEX (Figure 2B).^[^ ^49^ ^]^ This nanocarrier provides a novel avenue for codelivery of osteogenic gene and drugs to synergistically enhance the osteogenesis of BMSCs.

Recently, great attentions have been paid to develop drug delivery nanosystem with collaborative appropriate osteoimmunomodulation and osteoinductive environment that is beneficial for osteogenesis.^[^ ^50^ ^]^ Ye and co‐workers constructed a BMP‐2‐loaded deliver nanovehicles using *m*SiO_2_ rods with large cone‐shaped mesopores as carriers for bone regeneration (Figure 2C).^[^ ^51^ ^]^ The unique cone‐shaped mesopores could effectively reduce the pro‐inflammatory reaction of macrophages, further improve osteogenesis of BMSCs in vivo. Liu et al. design a multibiologic delivery nanosystem to in situ release IL‐2/TGF‐*β* and miR‐10a to locally recruit T cells and induce their differentiation into Tregs, providing potential opportunities for future bone immune and regenerative therapies (Figure 2D).^[^ ^52^ ^]^

### 3.2 Trace Elements for Regulating Bone Metabolism

**Figure 3:**
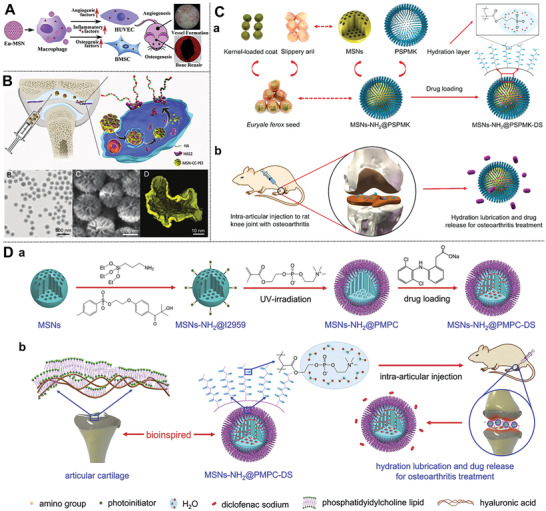
A) Schematic illustration of Eu‐doped MSNs. Reproduced with permission.^[^ ^59^ ^]^ Copyright 2017, Elsevier. B) Scheme of the cellular delivery of HAS2 to synoviocytes using MSN‐CC‐PEI nanoparticles. Reproduced with permission.^[^ ^71^ ^]^ Copyright 2019, WILEY‐VCH GmbH. C) Scheme of the design inspiration and treatment of OA by MSNs‐NH_2_@PSPMK‐DS. Reproduced with permission.^[^ ^74^ ^]^ Copyright 2019, WILEY‐VCH GmbH. D) Scheme of preparation and synergetic treatment of MSNs‐NH_2_@PSPMK nanoparticles. Reproduced with permission.^[^ ^76^ ^]^ Copyright 2020, Elsevier.

Besides bisphosphonate compounds, a multitude of trace elements, such as zinc (Zn),^[^ ^53^ ^]^ copper (Cu),^[^ ^40^, ^54^ ^]^ iron (Fe),^[^ ^55^ ^]^ magnesium (Mg),^[^ ^56^ ^]^ strontium (Sr),^[^ ^10^, ^40^ ^]^ gold (Au),^[^ ^57^ ^]^ silica (Si),^[^ ^58^ ^]^ manganese (Mn), europium (Eu),^[^ ^59^ ^]^ and gadolinium (Gd),^[^ ^12b^ ^]^ have important effects on regulating bone metabolism in the process of bone mineral, which was also widely introduced to the materials with osteoinductive potential to substitute either phosphate or calcium in the crystal lattice.^[^ ^60^ ^]^ In 1970, Carlisle reported that the Si element might have a significant influence on the initiation of preosseous tissue mineralization.^[^ ^16^ ^]^ Inspired by this, Wu and co‐workers^[^ ^58^ ^]^ synthesized a biodegradable and drug‐loaded MSNs, which could not only effectively release Si ions but also deliver functional hypoxia inducing therapeutic drug (e.g., dimethyloxaloylglycine) to induce the osteogenic differentiation and angiogenesis of *h*BMSCs. Additionally, previous reports demonstrated that some therapeutic ions, such as Sr ion, Mg ion, Zn ion, and Cu ion, play a crucial role in inducing the osteogenic and angiogenic differentiation of stem cells.^[^ ^61^ ^]^ Inspired by the idea that trace elements regulate bone metabolism, He's group synthesized a series of novel trace elements‐doped *m*HAP microspheres with drug delivery ability and enhanced bone regeneration through a microwave‐hydrothermal method. For example, they reported a hypoxia‐mimicking Cu‐doped *m*HAP microspheres using Doxorubicin (DOX) as a model drug.^[^ ^62^ ^]^ Accumulating evidence shows that *m*BGs with different therapeutic ions have attracted extensive interest in bone regeneration owing to their various merits such as favorable effects on stimulating osteogenesis/angiogenesis and excellent antibacterial ability. A Cu‐modified binary SiO_2_‐CaO *m*BG was designed via a one‐pot ultrasound‐assisted sol–gel method for remarkable bioactive behavior and antibacterial effect.^[^ ^40c^ ^]^ Similarly, a novel biomimetic DOX‐loaded Zn‐doped *m*HAP microspheres scaffold was synthesized, improving the osteoinductive potential and drug delivery.^[^ ^53^ ^]^ Yu and co‐workers reported novel Sr‐doped amorphous *m*CaP nanoparticles (Sr‐*m*CaPs) using vancomycin as antibiotics.^[^ ^10^ ^]^ The Sr‐*m*CaPs using fructose 1,6‐bisphosphate as organic phosphorus source display a more effective antibacterial capability than the pure *m*HAP nanorods using Na_2_HPO_4_·12H_2_O. Moreover, in order to prove the importance of Sr incorporation in enhancing bone regeneration, this work further introduced Sr‐*m*CaPs into collagen (Coll) through a freeze‐drying method to obtain a novel mimic scaffold. Results show that the tissue‐engineer scaffold significantly enhanced new bone formation in vivo at 8 weeks postimplantation as compared to without Sr‐doping. Wu and co‐workers reported hypoxia‐inducing Cu‐doped MSNs for Cu and Si ions dual‐delivery, endowing MSNs osteoimmunomodulatory properties.^[^ ^54^ ^]^ Interestingly, they successfully fabricated an immune‐modulating osteogenesis and angiogenesis agent based on in situ Eu‐doped MSNs (**Figure** **3**A).^[^ ^59^ ^]^ Compared with pure MSNs, the Eu/Au‐doped agent effectively upregulates the secretion of the proinflammatory/osteogenic cytokines and further activates the osteogenic/angiogenic differentiation, inducing favorable immune response of macrophages.^[^ ^57^ ^]^ Qin's group reported Mg and vitamin C supplementation for preventing steroid‐associated osteonecrosis in rats.^[^ ^56^ ^]^ Collectively, the strategy of co‐delivery both stimulatory ions and functional cargo provides a novel avenue to design a multifunctional cargo delivery system for bone regeneration or osteoinflammation.

### 3.3 Enzyme/Gene Delivery for Suppressing Pathogenic Gene Expressions

Poly(lactic‐*co*‐glycolic acid) (PLGA)/MSNs organic–inorganic hybrid MNs were prepared by Tasciotti's group for the dual‐delivery of bovine serum albumin (BSA) and the multiple porous silicon particles within the PLGA matrix.^[^ ^63^ ^]^ The released Si ion could neutralize the acidic pH because of the PLGA degradation by‐products,^[^ ^64^ ^]^ and decelerate the degradation of BSA.^[^ ^65^ ^]^ Thus, this nanocarrier significantly reduces inflammation and modulates bone biomineralization in bone tissue engineering.

Gene therapeutics (e.g., si‐RNA, DNA) play an essential role in inhibiting pathogenic gene expressions.^[^ ^66^ ^]^ Amino‐silanes and polycation polymers (e.g., Poly‐L‐lysine, PLL) are commonly utilized for chemical agents to provide polycations on the surfaces of MSNs via electrostatic interactions.^[^ ^67^ ^]^ In the early 2000s, Kneuer et al. first developed amino‐modified MSNs nanovehicle for gene delivery and protecting plasmid DNA.^[^ ^68^ ^]^ Indeed, previous studies have proved that large pore MSNs were more effective gene delivery nanovehicles than those with a small pore.^[^ ^69^ ^]^ Qiao and co‐workers designed a novel PLL‐modified MSNs with a large cubic mesostructured pore of ≈11.8 nm for gene delivery by dual templates (F127 and FC‐4) methods.^[^ ^70^ ^]^ After modification with PLL, a sufficient binding affinity, and strong delivery of MSNs for negatively charged nucleic acids (oligo‐DNA‐Cy3) was observed in osteosarcoma cancer cells as a comparison with pure and amino‐functionalized MSNs.

The introduction of external hyaluronic acid (HA) has been regarded as a typical strategy for the treatment of OA, but still meets some obstacles such as lasted for short time and required multiple injections. As a consequence, to promote the secretion of endogenous HA with a high molecular weight in joints HA, a novel one‐shot therapy strategy was necessary for the treatment of OA. It meets a challenge to improve the level of hyaluronan synthase type 2 (HAS2), the mainly generated enzyme of endogenous HA with high molecular weight inside synoviocytes. Based on this, Long and co‐workers^[^ ^71^ ^]^ first developed biodegradable MSNs with a core‐cone structure for the efficient intracellular delivery pathway of HAS2 in vitro and in vivo (Figure 3B). Notably, this nanoplatform significantly increased the yield of endogenous HA and effectively relieves the synovial inflammation, further providing an alternative and convenient approach for OA management.

### 3.4 Superlubricated Drugs for Inhibiting OA

As for the treatment of OA, synergetic therapy combining both lubrication and anti‐inflammatory drug intervention has been regarded as a promising nonsurgical strategy.^[^ ^72^ ^]^ In this regard, more efforts should be paid to design a facile strategy to construct superlubricated drug (e.g., polyelectrolyte polymer)‐loaded MSNs based on hydration lubrication mechanism without introducing toxic catalysts.^[^ ^73^ ^]^ Yan and co‐workers fabricated a facile superlubricated drug‐loaded MSNs through a low‐toxic photopolymerization method (Figure 3C).^[^ ^74^ ^]^ By the introduction of polyelectrolyte polymer, poly(3‐sulfopropyl methacrylate potassium salt) (PSPMK) on the surface of MSNs‐NH_2_, endows nanocarrier (MSNs‐NH_2_@PSPMK) synergetic capability of enhanced lubrication because of the tenacious hydration layers formation surrounding the negative charges and effective controlled drug delivery and in turn, the encapsulation of anti‐inflammatory drug (diclofenac sodium) improved its lubrication effects. Collectively, the above nanoplatform significantly protects the chondrocytes from degeneration, and thus, inhibits further growth of OA both in vitro and in vivo.

From the viewpoint of two interventions for OA in clinical, intra‐articular injection of HA cannot biomimic the hydration lubrication mechanism, and the oral administration of anti‐inflammatory drugs (e.g., glucosamine) is extremely hard to get to the joint without enough blood vessels. Hence, it was of vital importance to create therapeutic strategies to synergetically enhance lubrication and sustained drug delivery.^[72,^ ^75^ ^]^ Inspired by this, Zhang and co‐workers synthesized biomimetic superlubricated drug delivery nanovehicles via photopolymerization of poly(2‐methacryloyloxyethyl phosphorylcholine) brush with the same zwitterionic charges on the surface of MSNs‐NH_2_ microspheres (Figure 3D).^[^ ^76^ ^]^ This nanosystem significantly improved lubrication ability and enabled drug sustained release, retarded the degradation of oxidative stress‐induced chondrocytes, and decreased the expression of the pain‐related gene, and thus inhibited the development of OA. Collectively, the superlubricity intra‐articularly injected drug‐loaded nanosystem opened up more possibilities for the treatment of OA.

## 4 Stimuli‐Responsive MNs as Drug Controlled Release Systems

In the nonspecific bone‐targeting drug delivery system, there are mainly physicochemical targeted delivery systems and passive targeted delivery systems. A passive targeted delivery system refers to the uptake and delivery of drug‐loaded nanocarriers into the bone by the monocyte‐macrophage system. It can make use of high permeability and retention (EPR) effects in tumor tissue to enable drug selective accumulation.^[^ ^77^ ^]^ The physicochemical targeted delivery system was strongly dependent on the tumor microenvironment, which are often featured with the weakly acidic conditions, vascular abnormalities, hypoxia, glucose deprivation, high lactate content, and local hyperthermia in bone tumor and inflammatory tissues.^[^ ^78^ ^]^ The features of tumor microenvironment are not conducive to bone tumor treatment because it could effectively promote the immunosuppression by some bone tumor cells such as M2 tumor‐associated macrophages, myeloid‐derived suppressor cells.^[^ ^79^ ^]^ Hence, Tumor microenvironment plays a crucial role in the design of therapeutic nanocarriers.^[^ ^80^ ^]^

The stimuli‐responsive MNs have received widespread attention in nonspecific bone‐targeting drug sustained and controlled delivery system based on special one or more stimulation responsiveness in vitro or in vivo, such as physical (i.e., light, ultrasound, magnetism, heat), chemical (e.g., pH, redox potential), and biological (i.e., specific proteins, nucleic acids, and biomolecules) stimulation. The unique stimulation enables the structure of loaded cargo, the affinity with target sites, the solubility, and interaction force of cargo or modifier molecular change, to avoid premature drug release, improving the drug release efficiency of the target site, and reducing adverse drug reactions. The construction of stimulus‐responsive drug delivery nanosystem can be divided into two types: one is that the MNs carrier inherently has stimulus‐response properties; the other is those based on modifying the surface of MNs carrier with stimulus‐responsive groups or molecules. Thus, in this review, the design, principle, and construction of stimulus‐responsive nanodrug delivery system based on MNs as the carrier for bone regeneration are summarized according to the response factors (e.g., pH, light, temperature, ultrasound, enzyme, and redox).

### 4.1 pH Response

The pH value of tumor site and inflammatory tissue, especially in centrosomes and lysosomes in bone‐related cells is generally low, even reached about 6 or lower.^[^ ^81^ ^]^ The change of pH value can trigger the cargo release from MNs. When the pH reaches a certain critical value, the type and quantity of the charge of the specific molecules on the carriers and the electrostatic force between them can alter. Furthermore, the expansions or detentions of pH‐sensitive MNs materials are very critical for dispersing or gathering them. The pH‐responsive functionalized MNs acted as nanodrug delivery systems possess intriguing advantages in controllable movement and penetration of cargo molecules. Great efforts have been paid to regulating the capping and uncapping of pH‐responsive MNs through searching for suitable end‐capping agents, including pH‐sensitive linkers, supramolecular nanovalves, polyelectrolytes, or acid‐decomposable nanoobjects (e.g., ZnO quantum dots, QDs).^[^ ^82^ ^]^

**Figure 4:**
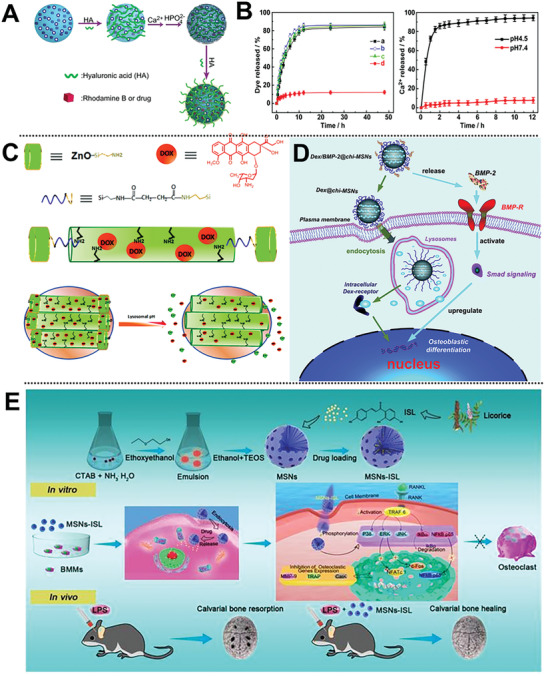
A) The biomineralization scheme of CaP‐HA‐coated MSNs. B) Release profiles of Ca^2+^ from MSNs in different pH. Reproduced with permission.^[^ ^83^ ^]^ Copyright 2020, Elsevier. C) Scheme of pH‐triggered release of DOX from ZnO@MSNs‐DOX. Reproduced with permission.^[^ ^82^ ^]^ Copyright 2012, American Chemical Society. D) Scheme of Dex/BMP‐2@chi‐MSNs on osteoblast differentiation. Reproduced with permission.^[^ ^84^ ^]^ Copyright 2015, the Royal Society of Chemistry. E) Scheme of synthesis and biological effects of MSNs‐ISL system. Reproduced under the terms of the Creative Commons License 4.0 (CC BY).^[^ ^89^ ^]^ Copyright 2019, The Authors, published by IVYSPRING.

Recent findings of the bone biomineralization mechanisms enable the molecular biomimetics of functional organic–inorganic hybrid nanocomposites. Qu et al. designed a natural hybrid capped MSNs by electrostatically absorbed polyanionic HA for the CaP deposition of heterogenous nucleation and response to pH‐stimulus (**Figure** **4**A).^[^ ^83^ ^]^ The attachment of HA layer on CaP surfaces enhanced the colloidal stability of nanovehicle and endows bone tumor cell with targeting capability. After receptor‐mediated endocytosis, the nanovehicle enables sustained release of anticancer drugs under acidic subcellular conditions (Figure 4B). Collectively, this strategy opens up a door for the biomineralization‐guided nanostructure self‐assembly.

Luminescent ZnO QDs have attracted increasing attention in pH‐triggered cargo release applications owing to the diverse merits of inexpensive, acid decomposability (pH <5.5), easy preparation, and excellent cytotoxic effects at target tumor sites. To inhibit the cationic premature drug (DOX) release, Zhu and co‐workers designed a facile method to construct a facile acid‐decomposable, DOX‐loaded luminescent ZnO QDs to cap MSNs with a diameter of 2.1 nm mesoporous nanochannels (Figure 4C).^[^ ^82^ ^]^ Notably, the ZnO QD lids (3–4 nm diameter) can be effectively dissolved in the acidic intracellular field of bone tumor cells, leading to the cargo release from the mesoporous channels of MSNs into the cytosol. Given the dual effection of blocking the end of ZnO QDs on MSNs and synergistic antitumor ability under acidic conditions, the ZnO@MSNs‐DOX composites provided a potential strategy toward the development of pH‐responsive anticancer cargo delivery that minimizes the drug toxicity.

To strengthen this synergistic osteoinductive effect and controlled dual‐delivery of a small drug, such as DEX and osteogenic protein (e.g., BMP‐2), a pH‐responsive chitosan‐functionalized MSN ensemble was synthesized by Liu's group (Figure 4D).^[^ ^84^ ^]^ This nanocarrier can quickly release bioactive BMP‐2 and then be efficiently endocytosed, and further enable controlled release of DEX in a weakly acidic condition in cells. Together, this nanoplatform significantly induces osteoblast differentiation, providing an effective strategy for clinical application feasibility by maintaining the bioactivity and optimizing dual cargo release mode.

OA, as a common inflammatory bone disease, featured with acidic environment and inflammation.^[^ ^85^ ^]^ Celastrol is an effective anti‐inflammatory and antioxidant drug as a possible treatment for OA. However, the low bioavailability and poor solubility make the clinical application of celastrol limited. Based on this, Jin et al. designed a highly soluble pH‐responsive nanosystem using hollow MSNs as nanocarrier to load celastrol and chitosan as protective outer layer for OA intra‐articular injection treatment.^[^ ^86^ ^]^ It is well known that the inflammation of bone tissue caused by pathogen exposure can induce excessive osteoclasts formation.^[^ ^87^ ^]^ Therefore, osteoclasts can be an effective target for cargo development against lytic bone diseases. Interestingly, some researches have revealed that isoliquiritigenin (2′,4′,4‐trithydroxychalcone, ISL), one of the flavonoids, can significantly inhibit osteoclastogenesis and modulate bone homeostasis.^[^ ^88^ ^]^ Despite this, ISL is still not enough for biomedical and clinical applications owing to its short elimination half‐life, poor solubility, and bioavailability. Peng and co‐workers for the first time developed licorice‐derived bioactive ISL‐loaded MSNs (MSNs‐ISL) as potential nanocarriers with good biocompatibility for preventing osteoclast‐mediated bone resorption (Figure 4E).^[^ ^89^ ^]^ The ISL release system favorably allows pH‐sensitive sustained drug release up to 60 h, and the cumulative release rate is higher (42.02% ± 1.63%) at pH 5.5 than that at pH 7.4. Moreover, MSNs‐ISL notably inhibited nuclear factor‐*κ*B ligand (RANKL)‐induced osteoclast formation in vitro, attenuated osteoclast activity, and protected against inflammatory bone destruction in vivo, which involves suppression of osteoclastogenetic expression as compared with pure ISL and MSNs.

**Figure 5:**
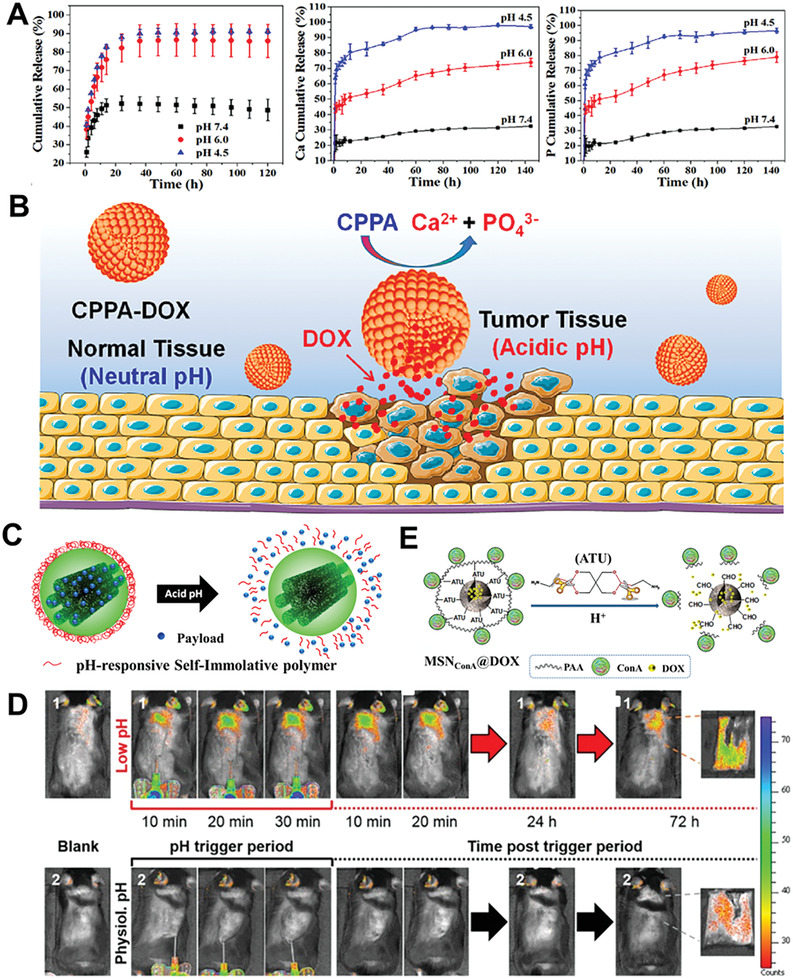
A) The DOX drug release profiles, dissolution performance of Ca, P elements, and B) Scheme of pH‐responsive release of CPPA/DOX nanosystem in vivo. Reproduced with permission.^[^ ^91^ ^]^ Copyright 2017, Elsevier. C) Scheme representation of pH‐responsive C1Sph‐SIP MNs; D) monitoring of Ru release from C1Sph‐SIP MNs. Reproduced with permission.^[^ ^9^ ^]^ Copyright 2020, American Chemical Society. E) Scheme of pH‐responsive behavior of the multifunctional tumor‐targeted MSN_ConA_@DOX nanodevice. Reproduced with permission.^[^ ^97^ ^]^ Copyright 2017, Elsevier.

Adenosine displays high influence on bone metabolism and osteogenic differentiation of BMSCs, providing a potential possibility for multiple myeloma‐associated bone disease treatment and prevention.^[^ ^90^ ^]^ Inspired by this, Tan and co‐workers developed a novel calcium phosphate‐phosphorylated adenosine (CPPA) MNs with a porous and hollow nanostructure.^[^ ^91^ ^]^ The DOX‐encapsulated CPPA delivery system shows high drug encapsulation efficiency (42.3%). The released rates of DOX and Ca, P ions are faster and the cumulative release amounts are higher from CPPA/DOX in a weak acid solution of solid tumors as compared to that in normal bone tissue (**Figure** **5**A).^[^ ^92^ ^]^ Furthermore, the pH‐responsive CPPA hybrid MNs display significant effects on stimulating osteogenic differentiation of *h*BMSCs, opening up the door for potential application in osteosarcoma (Figure 5B).^[^ ^93^ ^]^

Vallet‐Regí’s group has successfully fabricated a series of MNs with self‐immolation polymers (SIP) as novel pH‐responsive gatekeepers for drug delivery.^[^ ^94^ ^]^ Among them, two mesoporous carbon nanoparticles (C1Sph‐SIP) that acted as drug nanocarriers were prepared via a nanocasting procedure and functionalized with a self‐immolating polyurethane bearing a *tert*‐butyloxycarbonyl moiety as end‐cap sensitive to changes in pH (Figure 5C).^[^ ^9^ ^]^ The pH‐responsiveness of the C1Sph‐SIP nanoparticles show that the model cargo (e.g., Ru dye) release takes place inside the bone tumor cell and a chemotherapeutic drug such as DOX release could trigger growth inhibition of human osteosarcoma cells (Figure 5D). Additionally, the hybrid C1Sph‐SIP nanovehicles show a rapid pH‐responsiveness to short‐term triggering within acidic conditions in vivo after 72 h. Herein, the integrity and suitability of the nanosystem was regarded as biocompatible and efficient smart cargo delivery nanocarriers. Polyacrylic acid (PAA), a pH‐sensitive polymer, has been skillfully utilized as a pore‐blocking agent of MSNs owing to its diverse merits of biocompatibility, the abundance of carboxylic groups prone to be functionalized, and easy to anchor to the surface of MSNs through an acid‐cleavable acetal linker.^[^ ^95^ ^]^ The plant lectin concanavalin A (ConA), a targeting ligand, can selectively recognize and internalize certain cell‐surface glycans to increase the selectivity of the nanocarrier toward bone tumor cells.^[^ ^96^ ^]^ Herein, Vallet‐Regí and co‐workers developed a novel multifunctional DOX‐loaded MSNs system utilized as an antiosteosarcoma nanosystem for the assembly of pH‐sensitive PAA capping layer grafted to MSNs and a targeting ConA grafted to PAA (Figure 5E).^[^ ^97^ ^]^ Compared with healthy preosteoblast cells (MC3T3‐E1), this nanocarrier significantly minimizes premature cargo release, and shows a higher internalization level into human osteosarcoma cells. This nanoplatform coordinately improves antitumor efficiency and reduces toxicity to normal cells, providing a novel pathway for targeted bone tumor therapy.

### 4.2 Photoresponse

The transferred energy of the photo as an electromagnetic wave to the irradiated object enables the chemical bonds, polarity, configuration, and the structure of chemical groups to change, thus triggering cargo release or ligand shielding. In 2006, Mayer and Heckel proposed a design concept of “optical switch,” namely, the photolysis reaction occurs to remove the photosensitive protection group on the surface of materials utilized for masking the target molecule (activation) after the photo‐sensitive molecule absorbed a certain wavelength of photons, exposed, and restored the original activity of the target molecule, to realize the purpose of photoresponsive cargo release.^[^ ^98^ ^]^

**Figure 6:**
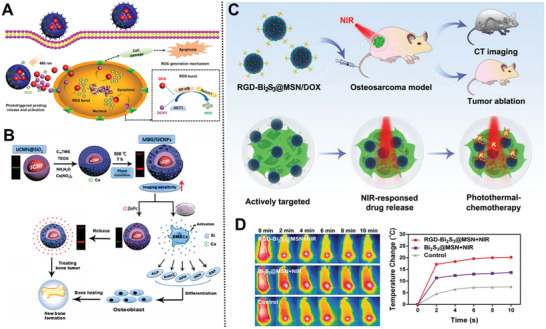
A) The intracellular mechanism of Ce6@ZA/MSN/DOX‐TK‐DOXY. Reproduced with permission.^[^ ^103^ ^]^ Copyright 2020, American Chemical Society. B) Schematic illustration of multifunctional UCNPs@SiO_2_@*m*SiO_2_/Ca nanosystems. Reproduced with permission.^[^ ^108^ ^]^ Copyright 2016, Springer. C) Treatment strategy of the smart RGD–Bi_2_S_3_@MSN/DOX nanosystems; D) in vivo therapy effect of different treatment groups. Reproduced with permission.^[^ ^115^ ^]^ Copyright 2018, WILEY‐VCH.

Phototherapies, such as photothermal therapy (PTT) and photodynamic therapy (PDT), have aroused great attention in bone tumor treatment owing to their high efficacy and negligible photoreactivity and toxicity.^[^ ^99^ ^]^ Treatment of bone tumors not only need to eliminate the residual bone tumor cells but also promote bone tissue regeneration. However, the penetration depth of near‐infrared (NIR) light restricted his application in deep tumorous bone defects.^[^ ^100^ ^]^ Chlorin e6 (Ce6), a second‐generation photosensitizer, possessed wide applicability for PDT due to its high chemical stability, high singlet oxygen (^1^O_2_) quantum yield, and neglectable dark toxicity.^[^ ^101^ ^]^ Great attention has been paid to the incorporation of PTT and Ce6 for treating tumorous bone defects, particularly in deeper tumors. Based on this, Wu's group successfully designed an NIR light‐triggered intelligent Mn‐doped Ce6‐loaded mesoporous bioactive glass (Mn‐MBG/Ce6) nanocontainer with high specific surface area via evaporation‐induced self‐assembly method for bone tumors treatment, especially for the deeper region.^[^ ^102^ ^]^ The incorporation of Mn into MBG not only imparted good PTT performance but also maintained well‐ordered mesopores. Interestingly, after loading Ce6 into the mesoporous channel of Mn‐MBG, this nanocarrier induced the NIR light‐triggered pulse release of Ce6 by a mild hyperthermia therapy, further enhancing the intracellular uptake of Ce6 by bone tumor cells. Interestingly, the controlled release of bioactive ions could accelerate bone regeneration initiated by NIR light. More importantly, even after a short term of synergistic PDT and PTT, the above‐mentioned nanocarriers still exhibited excellent biocompatibility and could promote long‐term osteanagenesis, especially in a deeper bone tumor. For effective combination of PDT and chemotherapy based on tumor‐associated reactive oxygen species (ROS) for osteosarcoma, Tu and co‐workers developed a multifunctional drug‐loaded nanoplatform based on MSNs‐modified bone‐targeting biphosphate moiety (**Figure** **6**A).^[^ ^103^ ^]^ This nanovehicle displays significant accumulation in osteosarcoma and long‐term retention in bone tumor sites. The release of DOXY accelerates the generation of ROS, and enables DOX/DOXY sustained release. The ROS amplification triggers long‐term high oxidative stress and increases the sensitivity toward osteosarcoma via chemotherapy, leading to enhance inhibition and apoptosis of bone tumor cells.

In addition to introducing photosensitizer into PTT for deep bone tumors, PDT combined with tunable multifunctional capacities of magnetic resonant imaging (MRI) and optical imaging (OI) has aroused great attention in a deeper tumorous bone application. TiO_2_ is one of the most efficient nanoplatforms for forming ROS photogeneration, whereas not suited for imaging because of its very faint fluorescence for OI and without magnetic.^[^ ^104^ ^]^ To overcome these drawbacks, many efforts have been devoted to explore diverse *m*TiO_2_ nanoparticles doped with rare‐earth elements such as Gd as luminescent nanomaterials for optical imaging probes in medicine.^[^ ^105^ ^]^ To improve photoluminescence performance, and enhance spin relaxation of MRI and photogeneration capacity of ROS, Iglic and co‐workers fabricated a novel Gd‐doped *m*TiO_2_ sub‐microspheres with the mesoporous channel.^[^ ^12b^ ^]^ ROS was generated only after photoactivation and a fully anticancer trace system for MRI was obtained just after 3 min irradiation‐incorporated PDT with OI–MRI with the biocompatible TiO_2_@10%Gd microspheres, thus paves the way to the simultaneous tumor bone cells imaging and treatment.

Upconversion nanoparticles (UCNPs) have received unprecedented attention in monitoring cargo release and PDT owing to their merits of emitting visible and NIR light when excited with NIR light, good photostability, deep penetration, negligible photodamage, and low autofluorescence.^[^ ^106^ ^]^ Many efforts have been devoted to introduce UCNPs (e.g., NaYF_4_:Yb/Er) with enhanced red emission output into biocompatible MNs to improve deeper penetration ability and monitor drug release in bone tumor imaging applications.^[^ ^107^ ^]^ In terms of the effect of UCNPs in optical imaging, Chang and co‐workers designed a multifunctional core–shell (UCNPs@SiO_2_@*m*SiO_2_/Ca) MBG/UCNP MNs through a layer‐by‐layer strategy for delivering anticancer drugs (e.g., zinc phthalocyanine), monitoring cargo release, and stimulating osteogenic differentiation of BMSCs (Figure 6B).^[^ ^108^ ^]^ Interestingly, the red luminescence could be extremely quenched by phthalocyanine on account of the overlaps among the maximum absorbance of the two. The incorporation of Ca^2+^ into the MNs could induce phase transformation and facilitated the red emission, improving fluorescence penetration for deeper bone tumorous imaging. Moreover, it also significantly stimulated apatite mineralization, enhanced proliferation, and bone‐related gene expression of BMSCs. Overall, this nanoplatform provides a new possibility for the therapy of malignant bone tumors.

Among NIR‐triggered nanoparticles, low‐cost bismuth‐doped nanoparticles (e.g., Bi_2_S_3_ nanorods,^[^ ^109^ ^]^ Cu_3_BiS_3_ nanodots,^[^ ^110^ ^]^ and hexahedron Cu_3_BiS_3_ nanoparticles^[^ ^111^ ^]^) are of great interest owing to their outstanding absorbance in the NIR region.^[^ ^112^ ^]^ Besides, bismuth‐doped nanoplatforms are expected to be served as novel X‐ray computed tomography (CT) contrast agents for multifunctional CT imaging and PTT theranostic because of their high atomic number.^[^ ^113^ ^]^ It is urgently needed to explore whether the PTT‐chemotherapy can alter the antiapoptotic Bcl‐2 protein‐dependent mitochondrial apoptosis pathway in a malignant tumor.^[^ ^114^ ^]^ Fabricated for the first time mesoporous silica‐coated bismuth sulfide MNs (RGD–Bi_2_S_3_@MSN) with uniform and discrete spherical morphology for malignant tumor diagnosis and the combined PTT‐chemotherapy (Figure 6C).^[^ ^115^ ^]^ The DOX‐loaded NPs exhibited an ultrahigh drug encapsulation efficiency (99.85%), strongly NIR‐triggered drug release, and distinct CT contrast imaging at the tumor site. After covalently conjugated to arginine‐glycine‐aspartic acid (RGD) peptide, the coordination of NPs and NIR irradiation ablates efficiently malignant osteosarcoma and prevents its recurrence (Figure 6D). Collectively, these above‐mentioned MNs provided a novel opportunity for the diagnosis of malignant bone tumor and the synergistic PTT‐chemotherapy.

### 4.3 Ultrasound Response

**Figure 7:**
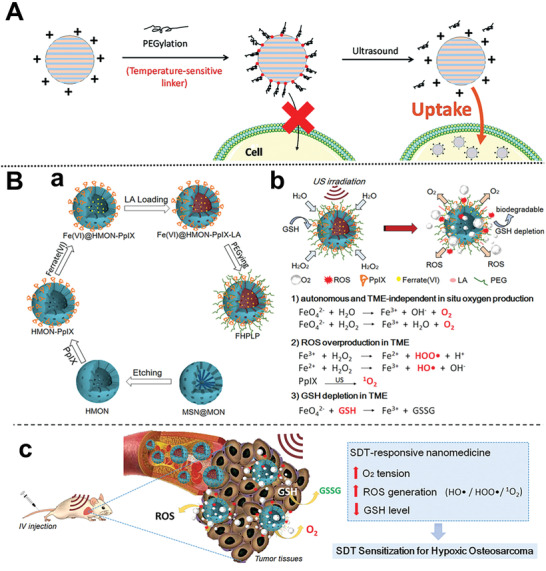
A) Scheme of MNs for cellular uptake by hierarchical ultrasound‐responsive positively charged MSN nanoplatforms. Reproduced with permission.^[^ ^120^ ^]^ Copyright 2018, Royal Society of Chemistry. B) Illustration of the fabrication and C) SDT sensitization of the nanosystem for hypoxic osteosarcoma. Reproduced with permission.^[^ ^121^ ^]^ Copyright 2019, WILEY‐VCH GmbH.

Sonodynamic therapy (SDT) has aroused great interest in the treatment of deep‐seated solid bone tumors owing to its integrated merits of noninvasive, high physical tolerance, and high body penetration depth, which enables ultrasound to trigger sonosensitizers for excess ROS generation^[^ ^116^ ^]^ and consequent oxidative cell damage.^[^ ^117^ ^]^ In this sense, Vallet‐Regí’s group developed a series of polymer‐grafted MSNs that acted as ultrasound‐responsive cargo carriers for bone tumor therapy.^[^ ^118^ ^]^ However, the cell uptake ability of MNs generally enhanced by employing positively charged moieties or internalization ligands (e.g., targeting agents) on MNs.^[^ ^119^ ^]^ Given this, they further developed a novel smart hierarchical ultrasound‐responsive positively charged MSN nanoplatforms capable of undergoing detachment of the PEG layer through an ultrasound‐induced temperature increase for bone tumor therapy (**Figure** **7**A).^[^ ^120^ ^]^ The ultrasound‐promoted nanoplatforms with positively charged surfaces drastically enhancing the cellular uptake and cytotoxic effect of topotecan‐loaded MNs in human osteosarcoma cells. Collectively, the functional MSNs can be triggered by remote ultrasound‐stimuli for cargo delivery and potential bone tumor therapy.

Besides, the efficacy of SDT can be limited by bone tumors hypoxia. Inspired by this, Hao and co‐workers developed an ultrasound‐triggered nanoplatform by incorporating ferrate(VI) and protoporphyrin IX into lauric acid‐assembled biodegradable hollow mesoporous organosilica nanocarriers (Figure 7B).^[^ ^121^ ^]^ The hyperthermia caused by US can induce the phase change of lauric acid, achieving US‐responsive control over the simultaneous release of oxygen and ROS, and in situ GSH depletion, which has a synergetic effect on sensitizing SDT to osteosarcoma. Hence, this nanosystem provides a novel avenue for potential application in hypoxic osteosarcoma treatment.

### 4.4 Other Responses

**Figure 8:**
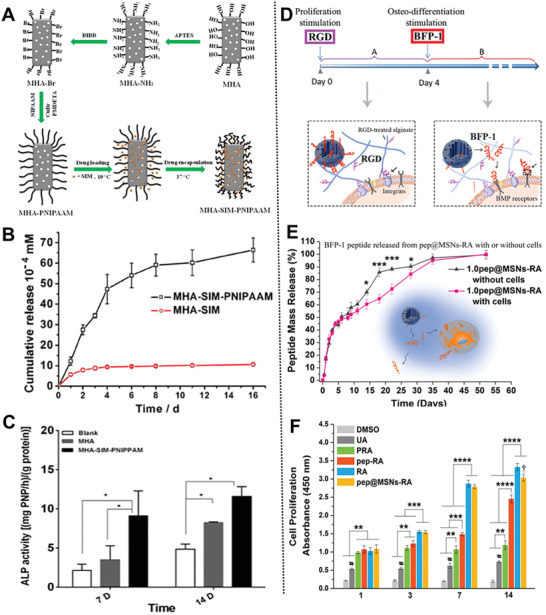
A) Scheme of the preparation progress of temperature‐responsive nanosystem; B) sustained release and C) osteogenesis promoting of MHA‐SIM‐PNIPAAM nanoparticles. Reproduced with permission.^[^ ^31a^ ^]^ Copyright 2016, Elsevier. D) Scheme of the dual‐peptide loaded nanosystem; E) BFP‐1 release behavior from pep@MSNs‐RA; F) proliferation of *h*MSCs. Reproduced with permission.^[^ ^123^ ^]^ Copyright 2018, Elsevier.

Shi and co‐workers developed a temperature‐responsive nanosystem based on simvastatin‐loaded *m*HAP modified by poly(*N*‐isopropylacrylamide) (PNIPAAM) for sustained osteogenic drug release and promoting cell attachment (**Figure** **8**A).^[^ ^31a^ ^]^ This nanovector shows almost sevenfold cumulative amount of simvastatin release, as well as better BMSCs proliferation and the osteogenic differentiation as compared with pure *m*HAP (Figure 8B,C). Besides, a magnetic‐responsive bone‐targeting nanovehicle based on ferrofluid‐containing iron‐oxide/silica/carbon nanoparticles (earth‐like particles) was successfully fabricated for brain and bone tumor‐targeting therapy though containing magnetic hyperthermia.^[^ ^122^ ^]^

Inspired by specific functional growth factors in different growth stages, Wei and co‐workers developed a sequentially triggered, alginate hybrid nanoplatform based on MSNs containing a laden bone‐forming peptide‐1 (BFP‐1) and the RGD sequence for inducing the differentiation of *h*MSCs (Figure 8D,E).^[^ ^123^ ^]^ BFP‐1 can be self‐captured via the cross‐linked networks between cell and peptide, which was induced by the ligands (RGD) binding to receptors on bone tumor cell, resulting in subsequent long‐term bone stimulation of hMSCs (Figure 8F). Collectively, this nanoplatform provided a time‐responsive dual‐peptide delivery strategy for osteo‐differentiation in bone tissue regeneration.

### 4.5 Multistimuli Response

**Figure 9:**
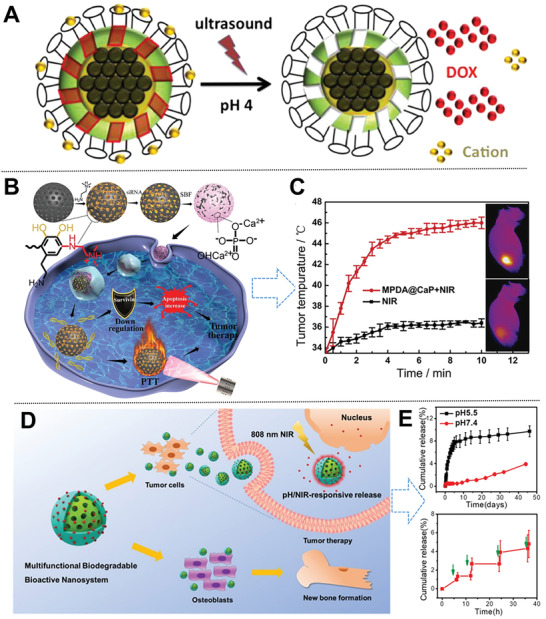
A) Scheme of multistimuli Fe_3_O_4_@SiO_2_@*m*SiO_2_@crown ethers. Reproduced with permission.^[^ ^124^ ^]^ Copyright 2013, American Chemical Society. B) Scheme on the preparation, PTT, and gene therapy of hybrid *m*PDA@CaP‐NIR nanosystem; C) in vivo antitumor efficacy of *m*PDA@CaP‐NIR MNs. Reproduced with permission.^[^ ^11a^ ^]^ Copyright 2019, Elsevier. D) Scheme of pH/NIR‐responsive BGs@*m*PDA‐DOX nanosystem; E) pH and NIR‐triggered stimuli‐responsive release from BGN@PDA. Reproduced with permission.^[^ ^126^ ^]^ Copyright 2020, American Chemical Society.

Core@shell magnetic mesoporous silica microspheres have played an essential role in applications of cargo delivery and osteocarcinoma cell imaging owing to the combined merits of superparamagnetic iron oxide core and *m*SiO_2_ shell.^[^ ^21^ ^]^ Lee et al. constructed an ultrasound, pH, and magnetically multistimuli responsive crown‐ether‐coated core/shell silica microspheres (Fe_3_O_4_@SiO_2_@*m*SiO_2_@crown ethers) as drug release systems (**Figure** **9**A).^[^ ^124^ ^]^ The integrated multistimuli responsive systems of PTT and siRNA display a significant influence on bone tumor treatment, whereas remains a great challenge for the integration and codelivery of gene payloads and PTT conversion agents.^[^ ^125^ ^]^ Based on the robust wet adhesion of marine mussels, Wang et al. developed a calcium phosphate (CaP)‐coated tertiary amines‐modified hybrid *m*PDA nanosystem with a high siRNA loading capacity of 10 wt% and PTT conversion efficiency of 37% (Figure 9B).^[^ ^11a^ ^]^ The successful construction of CaP coating on the cationic surface of *m*PDA via biomineralization not only prohibits the premature release of siRNA, yet underwent biodegradation at low pH value subcellular conditions. Compared with monotherapy, higher therapeutic efficiencies were demonstrated after combined with the efficient knocking down (65%) of the inhibitor of apoptosis protein (e.g., survivin) and PTT ablation. This nanoplatform smartly opened a novel door toward the utilization of bioadhesive surfaces and biomineralization coating for overcoming the bottlenecks of combined photothermal and gene therapy in a facile and simple process. Lei and co‐workers successfully developed a multifunctional biodegradable antibacterial nanosystem based on bioactive monodispersed bioactive glass nanoparticle (BGs) coated with mussel‐inspired *m*PDA for enhancing bone tissue regeneration and tumor PTT‐chemotherapy (Figure 9C).^[^ ^126^ ^]^ After encapsulated DOX, this nanosystem shows an on‐demand pH/NIR‐triggered cargo sustained release and antibacterial ability for enhanced bone tumor chemotherapy. Besides, this nanosystem significantly induced the osteogenesis of osteoblasts in vitro and possessed excellent bone regeneration in vivo. This bioactive multifunctional nanoplatform opened up a door for bone tumor‐resulted bone tissue regeneration therapeutic strategies.

## 5 MNs for Specific Bone‐Targeted Therapy

As compared with passive targeting, specific bone‐targeted therapy could make up for its shortcomings in various conditions such as the degree of different blood supply, tumor type, and anatomic location, especially in the bone tissue with less blood supply. It can be divided into two categories: one is to target specific bone‐related cells such as osteoclasts, osteoblasts, and BMSCs; the other is to target HAP, the inorganic matrix of bone tissue.^[^ ^127^ ^]^ The majority of bone tumor‐targeting treatment is focused on MNs modified with bisphosphonates, tetracycline, and bone affinity peptides (e.g., monoclonal antibodies, complements), polypeptides, nucleic acids as bone targeting group.^[^ ^128^ ^]^

### 5.1 Bisphosphonate for Inhibiting Bone Resorption

**Figure 10:**
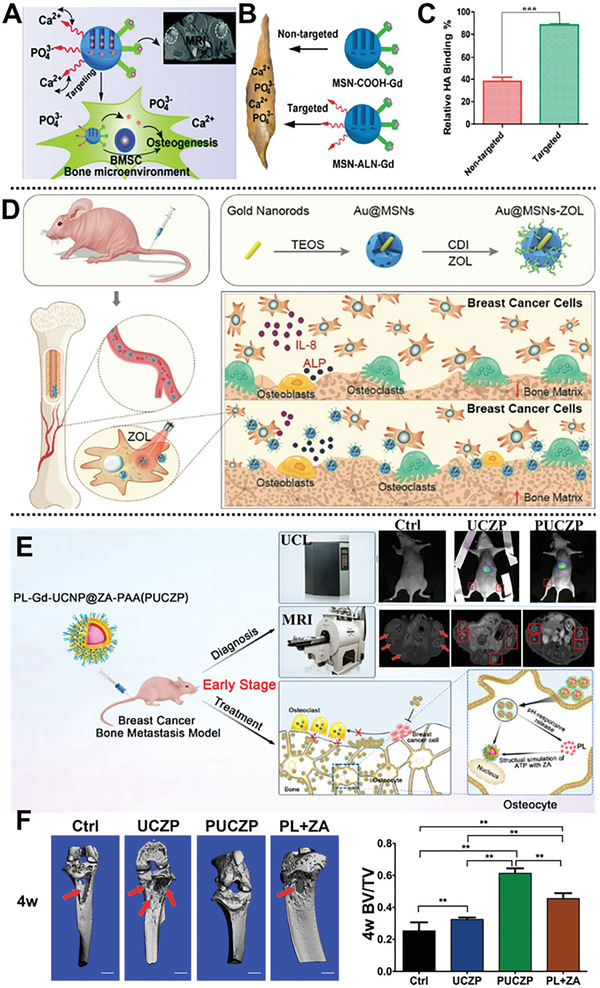
A) The macropinocytosis pathway of MSN‐ALN‐Gd nanovehicle; B) a diagram of nontargeted and targeted carriers binding to bone chips; C) the relative HA binding efficiency. Reproduced with permission.^[^ ^131^ ^]^ Copyright 2017, Royal Society of Chemistry. D) Au@MSNs combine ZOL and PTT for treating breast cancer bone metastasis. Reproduced with permission.^[^ ^133^ ^]^ Copyright 2019, American Chemical Society. E) Scheme of diagnosis and treatment of bone‐targeting PUCZP; F) theranostic effects of PUCZP against early bone metastasis. Reproduced with permission.^[^ ^137^ ^]^ Copyright 2017, American Chemical Society.

Bisphosphonates, such as etidronate, ibandronate, zoledronate (ZOL), alendronate (ALN), have been proved to be effective inhibitors of bone resorption via high affinity with solid CaP in bone matrix, for the treatment of bone metabolic diseases, osteoporosis in postmenopausal women, Paget's disease, and malignant tumors, such as multiple myeloma, malignant hypercalcemia, and prostate cancer.^[^ ^129^ ^]^ For example, Li et al. reported ibuprofen‐loaded *m*HAP nanoparticles for drug delivery, which was fabricated using CTAB as a template and functionalized by ALN.^[^ ^29a^ ^]^ Compared with pure *m*HAP, this functionalized *m*HAP indeed improved the drug loading or slowed down the release rate owing to the ionic interaction between –NH_3_^+^ on the matrix and –COO^–^ belongs to ibuprofen.^[^ ^130^ ^]^ Ren et al. fabricated a traceable and bone‐targeted/MRI dual‐functional sustained delivery nanocarrier based on walnut kernel‐like gadolinium‐labeled luminescent MSNs anchored by ALN using the soft‐template method for BMSCs homing and enhanced osteogenic differentiation (**Figure** **10**A).^[^ ^131^ ^]^ Meanwhile, the nanocarrier effectively stimulates the differentiation of BMSCs to osteoblast, and monitors the biodistribution in vivo through MRI ability.

ZOL has been employed as a bone‐targeting osteolysis inhibitor and bone mineral density enhancer owing to its strong affinity for bone rather than other tissues. Sun et al. fabricated a DOX‐encapsulated MSN anchored by ZOL for targeting bone matrix.^[^ ^132^ ^]^ This nanocarrier entered into A549 cells to realize effective intracellular DOX release. Moreover, it significantly decreased bone tumor cell migration in vitro. Furthermore, Zhang group^[^ ^133^ ^]^ created a ZOL‐conjugated nanocarrier using gold nanorods enclosed inside MSNs (Au@MSNs‐ZOL) for simultaneous bone‐targeting capacity and inhibiting osteoclast differentiation (Figure 10D). The combination of Au@MSNs‐ZOL and PTT, triggered by NIR irradiation, inhibited bone tumor growth, relieving bone resorption in vivo by inducing apoptosis in bone tumor cells. This coordination of ZOL and PTT toward nanocarrier opens up a door for breast cancer bone metastasis treatment.

Previously, a combination of ZOL with plumbagin (5‐hydroxy‐2‐methyl‐1,4‐naphthoquinone), has been demonstrated by Tang group to significantly suppress cancer and synergistically alleviates cancer‐induced osteoclastogenesis repression.^[^ ^134^ ^]^ UCNPs was of great interest due to their unique merits such as deep light penetration depth, good chemical/photostability as well as low photodamage to biosubstances.^[^ ^135^ ^]^ Interestingly, they further developed an UCNPs@MSNs doped with gadolinium(III), a common clinical contrast agent in T1‐MRI, for bone cancer multimodal synergetic therapy,^[^ ^136^ ^]^ whereas the drug burst release seriously limits its application. To solve this bottleneck, they creatively constructed a multifunctional bone‐targeting theranostic nanoplatform with an UCNPs@SiO_2_ core, gadolinium(III)‐doped ZOL‐anchored MSNs shell, a pH‐responsive plumbagin release, and poly(acrylic acid) (PAA) utilized for capping UCNPs in acidic conditions for coordinative diagnosis and treatment of early bone metastasis (Figure 10E).^[^ ^137^ ^]^ With the cooperation of plumbagin and ZOL, this nanoplatform can accurately target osteocytes to release plumbagin with pH‐responsiveness, synergistically inhibiting osteocytic RANKL and sclerostin expression. More importantly, tumorogenesis and osteoclastogenesis can both be attenuated. Inspiringly, it reveals that ZOL not only binds to activated HAP but also effectively targets osteocytes in vivo to regulate bone formation (Figure 10F). Therefore, this nanoplatform helps to pave a novel avenue on osteocyte‐targeting therapy for early bone metastasis, expanding the application of UCNPs to osteoporosis, OA, etc.

### 5.2 Bone Affinity Peptides for Therapeutic Osteosarcoma Cell Target

**Figure 11:**
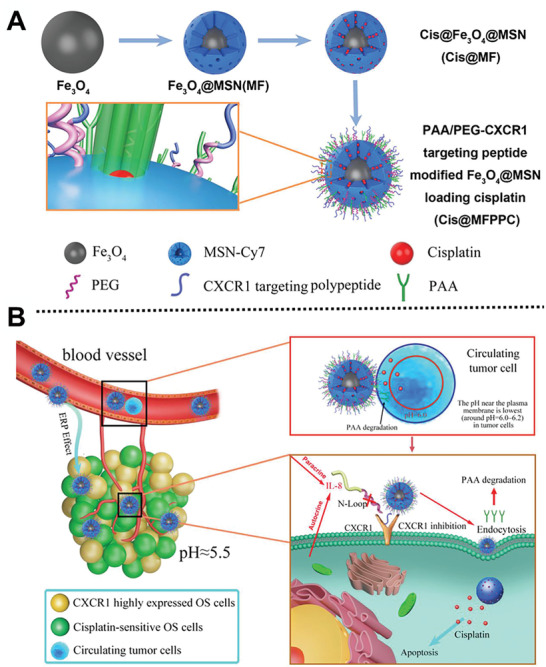
A) Scheme of synthesis process of Cis@MFPPC nanocarriers; B) scheme of antitumor mechanisms of Cis@MFPPC. Reproduced with permission.^[^ ^142^ ^]^ Copyright 2019, WILEY‐VCH GmbH.

Bone metastasis and chemotherapy resistance are two key obstacles to the complete eradication of osteosarcoma (e.g., breast cancers, prostate).^[^ ^138^ ^]^ Biomarkers on bone metastatic tumor cells were usually selected to be as therapeutic osteosarcoma cell targets. Bone metastatic breast cancers can be specifically targeted by combined with a monoclonal antibody (e.g., trastuzumab) against human epidermal growth factor receptor‐2 (HER2), a protooncogene overexpressed on up to 30% of breast cancers.^[^ ^139^ ^]^ Similarly, bone metastatic prostate cancer cells could be targeted by binding specific aptamers against prostate‐specific membrane antigen, which is overexpressed on them.^[^ ^140^ ^]^ CXCR1 is a specific receptor for IL‐8, and in turn, the N‐loop sequence of IL‐8 is of vital importance to bind to CXCR1, which played an essential role in bone metastasis and chemotherapy resistance.^[^ ^141^ ^]^ Inspired by mimicking the structure of IL‐8 to design a CXCR1 targeting peptide for targeting osteosarcoma circulating tumor cell (CTC), Tang group creatively designed a pH‐sensitive cisplatin‐loaded Fe_3_O_4_@MSNs linked with PEG‐linked CXCR1 and PAA as a detecting nanosystem, in order to inhibit the IL‐8/CXCR1 pathway and enhance the cisplatin sensitivity of CTCs through MRI and in vivo imaging system (**Figure** **11**).^[^ ^142^ ^]^ This nanosystem can inhibit osteosarcoma growth and pulmonary metastasis. This study possesses great promise for the clinical application of CXCR1 as a synergistic therapeutic osteosarcoma target and cargo delivery nanovector precise.

### 5.3 Aptamer for Reducing Osteoblast Differentiation by Inhibiting Specific Signaling Pathway

**Figure 12:**
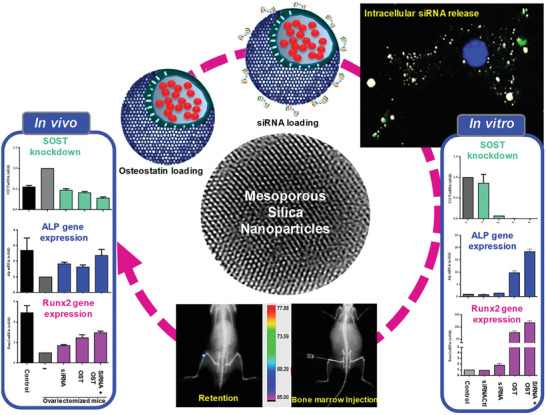
Scheme of the MSNs@osteostatin/siRNA nanosystem for osteoporosis treatment. Reproduced with permission.^[^ ^144^ ^]^ Copyright 2019, American Chemical Society.

The C‐terminal fragment of parathyroid hormone‐related protein (107−111) (osteostatin) has been regarded as an effective osteoclast inhibitor in osteoporosis.^[^ ^143^ ^]^ Silencing SOST gene with a specific small interfering RNA (siRNA) in osteocytes reduces osteoblast differentiation, and could overcome the immune response limitation. However, it is urgently necessary to design an effective nanovehicle for siRNA, to make up for deficiencies in very short half‐life and poor transfection capacity through cell membranes. In this aspect, Vallet‐Regí’s group^[^ ^144^ ^]^ designed creatively cationic polymers (poly‐(ethylenimine), PEI)‐modified MSNs with high loading capacity as nanovehicles to transport SOST siRNA and osteogenic peptide osteostatin to cells, stimulating osteoblastic cell growth and differentiation (**Figure** **12**). The coordination of SOST siRNA and the osteogenic peptide significantly increased markers expression of osteogenic differentiation. Taken together, this system will be a promising alternative for co‐delivery of gene therapy in osteoporosis treatment.

## 6 Conclusions and Outlook

This review discusses in‐depth the recent progress in the design and application of various MNs such as *m*SiO_2_‐based, *m*CaPs, *m*HAP, *m*PDA in bone disease diagnosis and treatment in the past decades. The precise properties and therapy and diagnosis mechanisms of diverse MNs were first systematically elucidated for bone‐related diseases, which are the foundation and play a key role in the design of MNs. Additionally, the relationship between MNs‐based multifunctional nanocarriers and bone regeneration, as well as the mechanisms among the distinct structure of MNs and cargo sustained release are summarized. Especially, the different therapy and diagnosis strategies were discussed and emphasized in depth, such as typical cargo sustained release, stimuli‐responsive (e.g., pH, photo, ultrasound) cargo controllable delivery, specific bone‐targeted therapy, and effective imaging agents for potential application in bone disease diagnosis and treatment. However, from the viewpoint of critical perspectives, the long‐term biosafety, and the elimination of metabolites after biodegradation, cargo sustained release, and targeted release ability should be further evaluated in practical clinical application.

Considering the above research bottlenecks, more methods and strategies are urgently needed to design novel MNs‐based nanocarriers for efficient bone disease treatment and diagnosis in practical clinical applications. First, more efforts should be paid to design functional MNs‐based nanocarriers and explore their sensitive‐response release mechanism, regulating the compositions and special structures such as cavity size, surface roughness and modification, core–shell or hollow structure, which can help improve the cargo loading and encapsulation amounts and increase cargo cumulative release. Second, based on the above‐mentioned diverse strategies, the integration of multifunctional diagnosis and treatment will be a promising development direction in the treatment of bone tumors and bone regeneration in the future. Notably, considerable efforts should be devoted to exploring the relationship among structure, cargo release, and diagnosis treatment from molecules level. Third, more attention should be paid to systematic therapy strategy which should be designed toward deeper bone tumors to improve limited penetration ability. A deeper understanding of bone targeting mechanism should also be further explored for finding more specific target ligands. With the ever‐strengthening collaboration of researchers from biomedicine and materials science, it is widely accepted that the structure–function relationship, diagnosis–treatment integration, and the potential stimulus‐responsive mechanism of MNs‐based nanocarriers are the most valuable and important research direction in the bone tumor application of cargo delivery engineering in the future.

## Conflict of Interest

The authors declare no conflict of interest.

## References

[1] A. G. Rodan , Science 2000, 289, 1508. 10968781 10.1126/science.289.5484.1508

[2] a) G. K. Shankar , S. Sampath , S. Muthusamy , M. J. Arlene , Mater. Sci. Eng., C 2019, 96, 941; 10.1016/j.msec.2018.11.081 30606606

[3] a) J. L. Harwood , J. H. Alexander , J. L. Mayerson , T. J. Scharschmidt , Orthop. Clin. N. Am. 2015, 46, 587; 10.1016/j.ocl.2015.06.011 26410647

[4] a) H. Song , M. Yu , Y. Lu , Z. Gu , Y. Yang , M. Zhang , J. Fu , C. Yu , J. Am. Chem. Soc. 2017, 139, 18247; 29151352 10.1021/jacs.7b08974

[5] M. Vallet‐Regí , P. Mora‐Raimundo , M. Manzano , AIMS Bioeng. 2017, 4, 259.

[6] a) M. Gisbert‐Garzaran , M. Manzano , M. Vallet‐Regi , Pharmaceutics 2020, 12, 83; 10.3390/pharmaceutics12010083 PMC7022913 31968690

[7] C. Qi , S. Musetti , L. H. Fu , Y. J. Zhu , L. Huang , Chem. Soc. Rev. 2019, 48, 2698. 31080987 10.1039/c8cs00489g

[8] Y. Zhou , M. Shi , J. R. Jones , Z. Chen , J. Chang , C. Wu , Y. Xiao , Int. Mater. Rev. 2017, 62, 392.

[9] M. Gisbert‐Garzaran , J. C. Berkmann , D. Giasafaki , D. Lozano , K. Spyrou , M. Manzano , T. Steriotis , G. N. Duda , K. Schmidt‐Bleek , G. Charalambopoulou , M. Vallet‐Regi , ACS Appl. Mater. Interfaces 2020, 12, 14946. 32141284 10.1021/acsami.0c01786 PMC7116326

[10] W. Yu , T.‐W. Sun , C. Qi , Z. Ding , H. Zhao , F. Chen , D. Chen , Y.‐J. Zhu , Z. Shi , Y. He , ACS Appl. Mater. Interfaces 2017, 9, 3306. 28068758 10.1021/acsami.6b12325

[11] a) Z. Wang , L. Wang , N. Prabhakar , Y. Xing , J. M. Rosenholm , J. Zhang , K. Cai , Acta Biomater. 2019, 86, 416; 30611792 10.1016/j.actbio.2019.01.002

[12] a) W. Luo , Z. Geng , Z. Li , S. Wu , Z. Cui , S. Zhu , Y. Liang , X. Yang , Int. J. Nanomed. 2018, 13, 7491; 10.2147/IJN.S177784 PMC6241695 30532535

[13] a) M. Colilla , M. Manzano , I. Izquierdo‐Barba , M. A. Vallet‐Regí , C. D. Boissiére , C. M. Sanchez , Chem. Mater. 2010, 22, 1821;

[14] A. K. Gaharwar , S. M. Mihaila , A. Swami , A. Patel , S. Sant , R. L. Reis , A. P. Marques , M. E. Gomes , A. Khademhosseini , Adv. Mater. 2013, 25, 3329. 23670944 10.1002/adma.201300584

[15] a) M. Manzano , M. Vallet‐Regí , Adv. Funct. Mater. 2019, 30, 1902634;

[16] E. M. Carlisle , Science 1970, 167, 279. 5410261 10.1126/science.167.3916.279

[17] K. Schwarz , Proc. Natl. Acad. Sci. U. S. A. 1973, 70, 1608. 4268099 10.1073/pnas.70.5.1608 PMC433552

[18] Y. Wang , Q. Zhao , N. Han , L. Bai , J. Li , J. Liu , E. Che , L. Hu , Q. Zhang , T. Jiang , S. Wang , Nanomedicine 2015, 11, 313. 25461284 10.1016/j.nano.2014.09.014

[19] a) L. Yu , Y. Chen , M. Wu , X. Cai , H. Yao , L. Zhang , H. Chen , J. Shi , J. Am. Chem. Soc. 2016, 138, 9881; 27441571 10.1021/jacs.6b04299

[20] a) Y. Jiang , S. Liu , Y. Zhang , H. Li , H. He , J. Dai , T. Jiang , W. Ji , D. Geng , A. A. Elzatahry , A. Alghamdi , D. Fu , Y. Deng , D. Zhao , Biomaterials 2017, 115, 9; 27871003 10.1016/j.biomaterials.2016.11.006

[21] Q. Yue , J. Sun , Y. Kang , Y. Deng , Angew. Chem., Int. Ed. 2020, 59, 2. 10.1002/anie.201911690 31593603

[22] H. Cheng , A. Chawla , Y. Yang , Y. Li , J. Zhang , H. L. Jang , A. Khademhosseini , Drug Discovery Today 2017, 22, 1336. 28487069 10.1016/j.drudis.2017.04.021 PMC5644493

[23] R. Narayan , U. Y. Nayak , A. M. Raichur , S. Garg , Pharmaceutics 2018, 10, 118. 10.3390/pharmaceutics10030118 PMC6160987 30082647

[24] a) A. G. Costa , N. E. Cusano , B. C. Silva , S. Cremers , J. P. Bilezikian , Nat. Rev. Rheumatol. 2011, 7, 447; 21670768 10.1038/nrrheum.2011.77

[25] a) X. Huang , L. Li , T. Liu , N. Hao , H. Liu , D. Chen , F. Tang , ACS Nano 2011, 5, 5390; 21634407 10.1021/nn200365a

[26] J. G. Croissant , K. S. Butler , J. I. Zink , C. J. Brinker , Nat. Rev. Mater. 2020, 5, 886.

[27] H. Zhang , D. R. Dunphy , X. Jiang , H. Meng , B. Sun , D. Tarn , M. Xue , X. Wang , S. Lin , Z. Ji , R. Li , F. L. Garcia , J. Yang , M. L. Kirk , T. Xia , J. I. Zink , A. Nel , C. J. Brinker , J. Am. Chem. Soc. 2012, 134, 15790. 22924492 10.1021/ja304907c PMC3505689

[28] S. Zhang , Z. Chu , C. Yin , C. Zhang , G. Lin , Q. Li , J. Am. Chem. Soc. 2013, 135, 5709. 23496255 10.1021/ja3123015

[29] a) D. Li , Y. Zhu , Z. Liang , Mater. Res. Bull. 2013, 48, 2201;

[30] a) J. R. Woodard , A. J. Hilldore , S. K. Lan , C. J. Park , A. J. W. Johnson , Biomaterials 2007, 28, 45; 16963118 10.1016/j.biomaterials.2006.08.021

[31] a) T. Wu , L. Tan , N. Cheng , Q. Yan , Y. F. Zhang , C. J. Liu , B. Shi , Mater. Sci. Eng., C 2016, 62, 888; 10.1016/j.msec.2016.01.012 PMC5995466 26952496

[32] P. Zhou , J. Wu , Y. Xia , Y. Yuan , H. Zhang , S. Xu , K. Lin , Int. J. Nanomed. 2018, 13, 4083. 10.2147/IJN.S158280 PMC6047624 30034234

[33] a) S.‐W. Ha , H. L. Jang , K. T. Nam , G. R. Beck , Biomaterials 2015, 65, 32; 26141836 10.1016/j.biomaterials.2015.06.039 PMC4508253

[34] X. Wang , W. Li , Nanotechnology 2016, 27, 225102. 27102805 10.1088/0957-4484/27/22/225102

[35] M. S. Kang , N.‐H. Lee , R. K. Singh , N. Mandakhbayar , R. A. Perez , J.‐H. Lee , H.‐W. Kim , Biomaterials 2018, 162, 183. 29448144 10.1016/j.biomaterials.2018.02.005

[36] a) C. Wu , J. Chang , Interface Focus 2012, 2, 292; 23741607 10.1098/rsfs.2011.0121 PMC3363021

[37] G. Miao , Z. Li , Y. Meng , J. Wu , Y. Li , Q. Hu , X. Chen , X. Yang , X. Chen , Adv. Powder Technol. 2019, 30, 1848.

[38] X. Yan , X. Huang , C. Yu , H. Deng , Y. Wang , Z. Zhang , S. Qiao , G. Lu , D. Zhao , Biomaterials 2006, 27, 3396. 16504289 10.1016/j.biomaterials.2006.01.043

[39] a) A. El‐Fiqi , J. H. Kim , H. W. Kim , ACS Appl. Mater. Interfaces 2015, 7, 1140; 25531645 10.1021/am5077759

[40] a) J. H. Lee , N. Mandakhbayar , A. El‐Fiqi , H. W. Kim , Acta Biomater. 2017, 60, 93; 28713017 10.1016/j.actbio.2017.07.021

[41] M. Vallet‐Regí , F. Balas , D. Arcos , Angew. Chem., Int. Ed. 2010, 46, 7548. 10.1002/anie.200604488 17854012

[42] M. Vallet‐Regi , A. Rámila , R. P. Del Real , J. Pérez‐Pariente , Chem. Mater. 2001, 13, 308.

[43] a) F. Balas , M. Manzano , P. Horcajada , M. Vallet‐Regí , J. Am. Chem. Soc. 2006, 128, 8116; 16787058 10.1021/ja062286z

[44] a) C. Przybylowski , T. Quinn , A. Callahan , M. Kaplan , A. Golding , C. Alesi , M. Ammar , C. E. Leblon , Y. Guo , X. Zhang , J. Mater. Chem. 2012, 22, 10672;

[45] a) X. Zhou , W. Feng , K. Qiu , L. Chen , W. Wang , W. Nie , X. Mo , C. He , ACS Appl. Mater. Interfaces 2015, 7, 15777; 26133753 10.1021/acsami.5b02636

[46] K. Dashnyam , G. Z. Jin , J. H. Kim , R. Perez , J. H. Jang , H. W. Kim , Biomaterials 2017, 116, 145. 27918936 10.1016/j.biomaterials.2016.11.053

[47] a) L. David , J. J. Feige , S. Bailly , Cytokine Growth Factor Rev. 2009, 20, 203; 19502096 10.1016/j.cytogfr.2009.05.001

[48] X. Zhao , Z. Li , H. Pan , W. Liu , M. Lv , F. Leung , W. W. Lu , Acta Biomater. 2013, 9, 6694. 23395816 10.1016/j.actbio.2013.01.039

[49] X. Zhou , Q. Zhang , L. Chen , W. Nie , W. Wang , H. Wang , X. Mo , C. He , ACS Biomater. Sci. Eng. 2019, 5, 710. 33405833 10.1021/acsbiomaterials.8b01110

[50] a) Z. Chen , J. Yuen , R. Crawford , J. Chang , C. Wu , Y. Xiao , Biomaterials 2015, 61, 126; 26001077 10.1016/j.biomaterials.2015.04.044

[51] C. Xu , L. Xiao , Y. Cao , Y. He , C. Lei , Y. Xiao , W. Sun , S. Ahadian , X. Zhou , A. Khademhosseini , Q. Ye , Nano Res. 2020, 13, 2323.

[52] Z. Liu , X. Chen , Z. Zhang , X. Zhang , L. Saunders , Y. Zhou , P. X. Ma , ACS Nano 2018, 12, 9785. 30141906 10.1021/acsnano.7b08976 PMC6205210

[53] W. Yu , T.‐W. Sun , C. Qi , Z. Ding , H. Zhao , S. Zhao , Z. Shi , Y.‐J. Zhu , D. Chen , Y. He , Int. J. Nanomed. 2017, 12, 2293. 10.2147/IJN.S126505 PMC5373825 28392688

[54] M. Shi , Z. Chen , S. Farnaghi , T. Friis , X. Mao , Y. Xiao , C. Wu , Acta Biomater. 2016, 30, 334. 26596565 10.1016/j.actbio.2015.11.033

[55] a) Y. P. Guo , L. H. Guo , Y. B. Yao , C. Q. Ning , Y. J. Guo , Chem. Commun. 2011, 47, 12215; 10.1039/c1cc15190h 21998826

[56] L. Z. Zheng , J. L. Wang , J. K. Xu , X. T. Zhang , B. Y. Liu , L. Huang , R. Zhang , H. Y. Zu , X. He , J. Mi , Q. Q. Pang , X. L. Wang , Y. C. Ruan , D. W. Zhao , L. Qin , Biomaterials 2020, 238, 119828. 32045781 10.1016/j.biomaterials.2020.119828 PMC7185815

[57] H. Liang , C. Jin , L. Ma , X. Feng , X. Deng , S. Wu , X. Liu , C. Yang , ACS Appl. Mater. Interfaces 2019, 11, 41758. 31610117 10.1021/acsami.9b16848

[58] M. Shi , Y. Zhou , J. Shao , Z. Chen , B. Song , J. Chang , C. Wu , Y. Xiao , Acta Biomater. 2015, 21, 178. 25910640 10.1016/j.actbio.2015.04.019

[59] M. Shi , L. Xia , Z. Chen , F. Lv , H. Zhu , F. Wei , S. Han , J. Chang , Y. Xiao , C. Wu , Biomaterials 2017, 144, 176. 28837959 10.1016/j.biomaterials.2017.08.027

[60] a) J. H. Beattie , A. Alison , Nutr. Res. Rev. 1992, 5, 167; 19094319 10.1079/NRR19920013

[61] C. Wu , J. Chang , J. Controlled Release 2014, 193, 282. 10.1016/j.jconrel.2014.04.026 24780264

[62] W. Yu , T.‐W. Sun , Z. Ding , C. Qi , H. Zhao , F. Chen , Z. Shi , Y.‐J. Zhu , D. Chen , Y. He , J. Mater. Chem. B 2017, 5, 1039. 32263882 10.1039/c6tb02747d

[63] D. Fan , E. De Rosa , M. B. Murphy , Y. Peng , C. A. Smid , C. Chiappini , X. Liu , P. Simmons , B. K. Weiner , M. Ferrari , E. Tasciotti , Adv. Funct. Mater. 2012, 22, 282.

[64] J. M. Chan , L. Zhang , K. P. Yuet , G. Liao , J. W. Rhee , R. Langer , O. C. Farokhzad , Biomaterials 2009, 30, 1627. 19111339 10.1016/j.biomaterials.2008.12.013

[65] A. J. Fulton , V. O. Kollath , K. Karan , Y. Shi , J. Appl. Phys. 2018, 124, 095701.

[66] a) B. Ozpolat , A. K. Sood , G. Lopez‐Berestein , J. Intern. Med. 2010, 267, 44; 20059643 10.1111/j.1365-2796.2009.02191.x

[67] a) T. Xia , M. Kovochich , M. Liong , H. Meng , S. Kabehie , S. George , J. I. Zink , A. E. Nel , ACS Nano 2009, 3, 3273; 19739605 10.1021/nn900918w PMC3900639

[68] C. Kneuer , M. Sameti , E. G. Haltner , T. Schiestel , H. Schirra , H. Schmidt , C. M. Lehr , Int. J Pharm. 2000, 196, 257. 10699731 10.1016/s0378-5173(99)00435-4

[69] a) F. Gao , P. Botella , A. Corma , J. Blesa , L. Dong , J. Phys. Chem. B 2009, 113, 1796; 19152258 10.1021/jp807956r

[70] S. B. Hartono , W. Gu , F. Kleitz , J. Liu , L. He , A. P. J. Middelberg , C. Yu , G. Q. M. Lu , S. Z. Qiao , ACS Nano 2012, 6, 2104. 22385282 10.1021/nn2039643

[71] H. Li , H. Guo , C. Lei , L. Liu , L. Xu , Y. Feng , J. Ke , W. Fang , H. Song , C. Xu , C. Yu , X. Long , Adv. Mater. 2019, 31, 1904535. 10.1002/adma.201904535 31549776

[72] G. Liu , M. Cai , F. Zhou , W. Liu , J. Phys. Chem. B 2014, 118, 4920. 24735439 10.1021/jp500074g

[73] a) J. Klein , Science 2009, 323, 47;19119205

[74] Y. Yan , T. Sun , H. Zhang , X. Ji , Y. Sun , X. Zhao , L. Deng , J. Qi , W. Cui , H. A. Santos , H. Zhang , Adv. Funct. Mater. 2019, 29, 1807559.

[75] S. Tao , S. Yulong , Z. Hongyu , Polymers 2018, 10, 513.

[76] H. Chen , T. Sun , Y. Yan , X. Ji , Y. Sun , X. Zhao , J. Qi , W. Cui , L. Deng , H. Zhang , Biomaterials 2020, 242, 119931. 32145507 10.1016/j.biomaterials.2020.119931

[77] H. Maeda , J. Drug Targeting 2017, 25, 781. 10.1080/1061186X.2017.1365878 28988499

[78] a) M. F. Chung , H. Y. Liu , K. J. Lin , W. T. Chia , H. W. Sung , Angew. Chem., Int. Ed. Engl. 2015, 54, 9890; 26136242 10.1002/anie.201504444

[79] a) A. Facciabene , X. Peng , I. S. Hagemann , K. Balint , A. Barchetti , L. P. Wang , P. A. Gimotty , C. B. Gilks , P. Lal , L. Zhang , G. Coukos , Nature 2011, 475, 226; 21753853 10.1038/nature10169

[80] a) G. B. Yang , H. Gong , X. X. Qian , P. L. Tan , Z. W. Li , T. Liu , J. J. Liu , Y. Y. Li , Z. Liu , Nano Res. 2014, 8, 751;

[81] E. S. Lee , Z. Gao , Y. H. Bae , J. Controlled Release 2008, 132, 164. 10.1016/j.jconrel.2008.05.003 PMC2695946 18571265

[82] F. Muhammad , M. Guo , W. Qi , F. Sun , A. Wang , Y. Guo , G. Zhu , J. Am. Chem. Soc. 2011, 133, 8778. 21574653 10.1021/ja200328s

[83] Z. Chen , Z. Li , Y. Lin , M. Yin , J. Ren , X. Qu , Biomaterials 2013, 34, 1364. 23140999 10.1016/j.biomaterials.2012.10.060

[84] Q. Gan , J. Zhu , Y. Yuan , H. Liu , J. Qian , Y. Li , C. Liu , J. Mater. Chem. B 2015, 3, 2056. 32262373 10.1039/c4tb01897d

[85] D. Chen , J. Shen , W. Zhao , T. Wang , L. Han , J. L. Hamilton , H. J. Im , Bone Res. 2017, 5, 16044. 28149655 10.1038/boneres.2016.44 PMC5240031

[86] T. Jin , D. Wu , X. M. Liu , J. T. Xu , B. J. Ma , Y. Ji , Y. Y. Jin , S. Y. Wu , T. Wu , K. Ma , J. Nanobiotechnol. 2020, 18, 94. 10.1186/s12951-020-00651-0 PMC7346635 32641147

[87] K. H. M. Yip , H. Z. Ming , T. F. Hao , J. H. Steer , J. Xu , J. Bone Miner. Res. 2010, 19, 1905.

[88] a) L. Zhu , H. Wei , Y. Wu , S. Yang , L. Xiao , J. Zhang , B. Peng , Int. J. Biochem. Cell Biol. 2012, 44, 1139; 22521613 10.1016/j.biocel.2012.04.003

[89] X. Sun , J. Zhang , Z. Wang , B. Liu , S. Zhu , L. Zhu , B. Peng , Theranostics 2019, 9, 5183. 31410209 10.7150/thno.33376 PMC6691588

[90] a) A. Mediero , B. N. Cronstein , Trends Endocrinol. Metab. 2013, 24, 290; 23499155 10.1016/j.tem.2013.02.001 PMC3669669

[91] Z. F. Zhou , T. W. Sun , F. Chen , D. Q. Zuo , H. S. Wang , Y. Q. Hua , Z. D. Cai , J. Tan , Biomaterials 2017, 121, 1. 28063979 10.1016/j.biomaterials.2016.12.031

[92] A. Tomida , T. Tsuruo , Anti‐Cancer Drug Des. 1999, 14, 169. 10405643

[93] a) C. Qi , Y. J. Zhu , B. Q. Lu , G. J. Ding , T. W. Sun , F. Chen , J. Wu , J. Mater. Chem. B 2014, 2, 8576; 32262216 10.1039/c4tb01473a

[94] M. Gisbert‐Garzaran , D. Lozano , M. Vallet‐Regí , M. Manzano , RSC Adv. 2017, 7, 132.

[95] a) R. Liu , Y. Zhang , X. Zhao , A. Agarwal , L. J. Mueller , P. Feng , J. Am. Chem. Soc. 2010, 132, 1500; 20085351 10.1021/ja907838s

[96] a) E. J. M. V. Damme , W. J. Peumans , A. Barre , P. Rougé , Crit. Rev. Plant Sci. 1998, 17, 575;

[97] M. Martinez‐Carmona , D. Lozano , M. Colilla , M. Vallet‐Regi , Acta Biomater. 2018, 65, 393. 29127069 10.1016/j.actbio.2017.11.007

[98] G. Mayer , A. Heckel , Angew. Chem., Int. Ed. 2006, 45, 4900. 10.1002/anie.200600387 16826610

[99] a) L. Cheng , C. Wang , L. Feng , K. Yang , Z. Liu , Chem. Rev. 2014, 114, 10869; 25260098 10.1021/cr400532z

[100] a) Y. Maruoka , T. Nagaya , K. Sato , F. Ogata , S. Okuyama , P. L. Choyke , H. Kobayashi , Mol. Pharmaceutics 2018, 15, 3634; 10.1021/acs.molpharmaceut.8b00002 PMC7400989 29450993

[101] a) Y. Zhang , L. He , J. Wu , K. Wang , J. Wang , W. Dai , A. Yuan , J. Wu , Y. Hu , Biomaterials 2016, 107, 23; 27598652 10.1016/j.biomaterials.2016.08.037

[102] Y. Liu , R. Lin , L. Ma , H. Zhuang , C. Feng , J. Chang , C. Wu , Appl. Mater. Today 2020, 19, 100578.

[103] F. Tong , Y. Ye , B. Chen , J. Gao , L. Liu , J. Ou , J. C. M. Van Hest , S. Liu , F. Peng , Y. Tu , ACS Appl. Mater. Interfaces 2020, 12, 34630. 32635715 10.1021/acsami.0c08992

[104] D. E. J. G. J. Dolmans , F. Dai , R. K. Jain , Nat. Rev. Cancer 2003, 3, 380. 12724736 10.1038/nrc1071

[105] H. B. Na , I. C. Song , T. Hyeon , Adv. Mater. 2009, 21, 2133.

[106] a) A. E. Guller , A. N. Generalova , E. V. Petersen , A. V. Nechaev , I. A. Trusova , N. N. Landyshev , A. Nadort , E. A. Grebenik , S. M. Deyev , A. B. Shekhter , Nano Res. 2015, 8, 1546;

[107] a) J. Liu , J. Bu , W. Bu , S. Zhang , L. Pan , W. Fan , F. Chen , L. Zhou , W. Peng , K. Zhao , J. Du , J. Shi , Angew. Chem., Int. Ed. 2014, 126, 4639; 10.1002/anie.201400900 24668766

[108] F. Wang , D. Zhai , C. Wu , J. Chang , Nano Res. 2016, 9, 1193.

[109] J. Liu , X. Zheng , Z. Gu , C. Chen , Y. Zhao , ACS Nano 2016, 12, 486.

[110] J. Liu , P. Wang , X. Zhang , L. Wang , D. Wang , Z. Gu , J. Tang , M. Guo , M. Cao , H. Zhou , ACS Nano 2016, 4587. 27014806 10.1021/acsnano.6b00745

[111] R. Dong , Y. Fang , J. Chae , J. Dai , Z. Xiao , Q. Dong , Y. Yuan , A. Centrone , X. C. Zeng , J. Huang , Adv. Mater. 2015, 27, 1912. 25605226 10.1002/adma.201405116

[112] J. W. Thomson , P. O'Brien , R. Klenkler , M. G. Helander , S. Petrov , Z.‐H. Lu , N. P. Kherani , A. Adronov , G. Ozin , Adv. Mater. 2010, 22, 4395. 20540105 10.1002/adma.201001349

[113] a) H. Lusic , M. W. Grinstaff , Chem. Rev. 2013, 113, 1641; 23210836 10.1021/cr200358s PMC3878741

[114] D. Narendra , J. E. Walker , R. Youle , Cold Spring Harbor Perspect. Biol. 2012, 4, 843. 10.1101/cshperspect.a011338 PMC3536340 23125018

[115] Y. Lu , L. Li , Z. Lin , M. Li , X. Hu , Y. Zhang , M. Peng , H. Xia , G. Han , Adv. Healthcare Mater. 2018, 7, 1800602. 10.1002/adhm.201800602 PMC6504251 30102469

[116] a) X. Qian , Y. Zheng , Y. Chen , Adv. Mater. 2016, 28, 8097; 27384408 10.1002/adma.201602012

[117] A. K. W. Wood , C. M. Sehgal , Ultrasound Med. Biol. 2015, 41, 905. 25728459 10.1016/j.ultrasmedbio.2014.11.019 PMC4362523

[118] J. L. Paris , M. V. Cabanas , M. Manzano , M. a. Vallet‐Regı´ , ACS Nano 2015, 9, 11023. 26456489 10.1021/acsnano.5b04378

[119] S. Wang , P. Huang , X. Chen , Adv. Mater. 2016, 28, 7340. 27255214 10.1002/adma.201601498 PMC5014563

[120] J. L. Paris , M. Manzano , M. V. Cabañas , M. Vallet‐Regí , Nanoscale 2018, 10, 6402. 29561558 10.1039/C8NR00693H

[121] J. Fu , T. Li , Y. Zhu , Y. Hao , Adv. Funct. Mater. 2019, 29, 1906195.

[122] V. M. Wu , E. Huynh , S. Tang , V. Uskokovic , Acta Biomater. 2019, 88, 422. 30711662 10.1016/j.actbio.2019.01.064

[123] Z. Luo , S. Zhang , J. Pan , R. Shi , H. Liu , Y. Lyu , X. Han , Y. Li , Y. Yang , Z. Xu , Y. Sui , E. Luo , Y. Zhang , S. Wei , Biomaterials 2018, 163, 25. 29452946 10.1016/j.biomaterials.2018.02.025

[124] S. F. Lee , X. M. Zhu , Y. X. Wang , S. H. Xuan , Q. You , W. H. Chan , C. H. Wong , F. Wang , J. C. Yu , C. H. Cheng , K. C. Leung , ACS Appl. Mater. Interfaces 2013, 5, 1566. 23402574 10.1021/am4004705

[125] a) Y. Zhang , K. Ren , X. Zhang , Z. Chao , Y. Yang , D. Ye , Z. Dai , Y. Liu , H. Ju , Biomaterials 2018, 163, 55; 29452948 10.1016/j.biomaterials.2018.02.019

[126] Y. Xue , W. Niu , M. Wang , M. Chen , Y. Guo , B. Lei , ACS Nano 2020, 14, 442. 31702885 10.1021/acsnano.9b06145

[127] E. J. Carbone , K. Rajpura , B. N. Allen , E. Cheng , W. H. Lo , Nanomedicine 2017, 13, 37. 27562211 10.1016/j.nano.2016.08.015

[128] X. Gao , L. Li , X. P. Cai , Q. Huang , J. R. Xiao , Y. Y. Cheng , Biomaterials 2021, 265, 120404. 32987273 10.1016/j.biomaterials.2020.120404

[129] a) M. J. Rogers , D. J. Watts , R. G. G. Russell , Cancer 2015, 80, 1652; 10.1002/(sici)1097-0142(19971015)80:8+<1652::aid-cncr15>3.0.co;2-z 9362432

[130] L. Jiang , X. Li , L. Liu , Q. Zhang , Int. J. Nanomed. 2013, 8, 1825. 10.2147/IJN.S44342 PMC3652563 23674894

[131] H. Ren , S. Chen , Y. Jin , C. Zhang , X. Yang , K. Ge , X.‐J. Liang , Z. Li , J. Zhang , J. Mater. Chem. B 2017, 5, 1585. 32263930 10.1039/c6tb02552h

[132] W. Sun , Y. Han , Z. Li , K. Ge , J. Zhang , Langmuir 2016, 32, 9237. 27531422 10.1021/acs.langmuir.6b02228

[133] W. Sun , K. Ge , Y. Jin , Y. Han , H. Zhang , G. Zhou , X. Yang , D. Liu , H. Liu , X. J. Liang , J. Zhang , ACS Nano 2019, 13, 7556. 31259530 10.1021/acsnano.9b00097

[134] a) H. Qiao , T. Wang , Z. Yu , X. Han , X. Liu , Y. Wang , Q. Fan , A. Qin , T. Tang , Cell Death Dis. 2016, 7, e2094; 26866274 10.1038/cddis.2016.11 PMC4849151

[135] W. Feng , C. Han , F. Li , Adv. Mater. 2013, 25, 5287. 23982981 10.1002/adma.201301946

[136] W. Fan , B. Shen , W. Bu , F. Chen , Q. He , Biomaterials 2014, 35, 8992. 25103233 10.1016/j.biomaterials.2014.07.024

[137] H. Qiao , Z. Cui , S. Yang , D. Ji , Y. Wang , Y. Yang , X. Han , Q. Fan , A. Qin , T. Wang , X. P. He , W. Bu , T. Tang , ACS Nano 2017, 11, 7259. 28692257 10.1021/acsnano.7b03197

[138] N. Federman , J. Chan , J. O. Nagy , E. M. Landaw , K. McCabe , A. M. Wu , T. Triche , H. Kang , B. Liu , J. D. Marks , C. T. Denny , Sarcoma 2012, 2012, 126906. 23024593 10.1155/2012/126906 PMC3447386

[139] a) L. Pourtau , H. Oliveira , J. Thevenot , Y. Wan , A. R. Brisson , O. Sandre , S. Miraux , E. Thiaudiere , S. Lecommandoux , Adv. Healthcare Mater. 2013, 2, 1420; 10.1002/adhm.201300061 23606565

[140] Z. Hao , W. Fan , J. Hao , X. Wu , G. Q. Zeng , L. J. Zhang , S. F. Nie , X. D. Wang , Drug Delivery 2016, 23, 864. 10.3109/10717544.2014.920059 24892627

[141] a) Q. Liu , A. Li , Y. Tian , J. D. Wu , Y. Liu , T. Li , Y. Chen , X. Han , K. Wu , Cytokine Growth Factor Rev. 2016, 31, 61; 27578214 10.1016/j.cytogfr.2016.08.002 PMC6142815

[142] X. G. Han , S. B. Yang , H. B. Mo , M. Q. Wang , F. Zhou , H. J. Li , H. Qiao , J. T. Mei , Y. J. Wang , Y. W. Cheng , X. Q. Liu , L. Du , Y. Dong , T. T. Tang , Adv. Funct. Mater. 2019, 29, 1902246.

[143] a) D. Lozano , L. Fernández‐de‐Castro , S. Portal‐Núez , A. López‐Herradón , P. Esbrit , Br. J. Pharmacol. 2015, 162, 1424; 10.1111/j.1476-5381.2010.01155.x PMC3058173 21175568

[144] P. Mora‐Raimundo , D. Lozano , M. Manzano , M. Vallet‐Regi , ACS Nano 2019, 13, 5451. 31071265 10.1021/acsnano.9b00241 PMC6588271

